# Synthesis of New Perhydropyrrolo[1,2-*a*]pyrazine Derivatives and Their Evaluation in Animal Models of Epilepsy

**DOI:** 10.3390/molecules191015955

**Published:** 2014-10-07

**Authors:** Maciej Dawidowski, Wojciech Lewandowski, Jadwiga Turło

**Affiliations:** 1 Department of Drug Technology and Pharmaceutical Biotechnology, Medical University of Warsaw, Banacha 1 Str., 02-097 Warszawa, Poland; E-Mail: jadwiga.turlo@wum.edu.pl; 2 Faculty of Pharmacy, Medical University of Warsaw, Banacha 1 Str., 02-097 Warszawa, Poland; E-Mail: lewandowski.w@o2.pl

**Keywords:** 2,6-diketopiperazine, pyrrolo[1,2-*a*]pyrazine, multicomponent reaction, Ugi reaction, U-5C-4CR, U-4C-3CR, anticonvulsant agent, antiseizure agent, antiepileptic agent

## Abstract

A series of novel stereochemically pure derivatives of the investigative broad-spectrum anticonvulsant ADD408003 was designed and synthesized. Five-center four-component (U-5C-4CR) and four-center three-component (U-4C-3CR) variants of Ugi reaction were used in the key step of the synthetic pathways. The compounds obtained were evaluated for the anticonvulsant activitiy in the maximal electroshock seizure (MES), subcutaneous Metrazole (scMET) and minimal clonic seizure (6 Hz) animal models of epilepsy. The efficacies of most derivatives in the 6 Hz model of pharmacoresistant partial seizures were markedly higher than in the ‘classical’ MES and scMET models. The most active compounds, **(4*R*,8a*R*)-3a**, and **(4*S*,8a*S*)-6** displayed median effective doses (ED_50_) of 47.90 and 126.19 mg/kg, respectively, for the 6 Hz test.

## 1. Introduction

According to epidemiological studies, epilepsy affects approximately 1% of the world’s population [1]. Although significant advances have been achieved in pharmacotherapy of this disorder, currently available anticonvulsant drugs (AEDs) produce satisfactory seizure control only in 60%–70% of patients. Moreover, their usage is often associated with disturbing side-effects which seriously limit quality of patients’ life. Thus, there is a substantial need for further development of novel, safer and more efficient AEDs [2,3,4,5,6].

**Figure 1:**
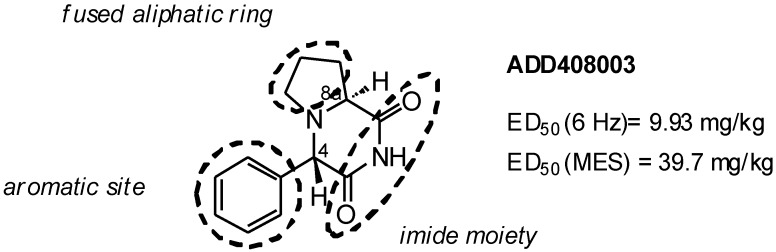
Structure of ADD408003.

In our recent reports, we described a structurally novel class of investigative anticonvulsants, derivatives of perhydropyrrolo[1,2-*a*]pyrazines [7]. Among them, compound ADD408003 (Figure 1) revealed high and broad activity in various preclinical animal models of epilepsy, including the models of pharmacoresistant epilepsy. A preliminary structure–activity relationship (SAR) study revealed that several structural patterns are necessary for the high anticonvulsant activity: the (*S*,*S*) absolute configuration on the stereogenic centers, the presence of the annulated pyrrolidine ring, the presence of imide moiety and the benzene ring in C-4 position of the pyrrolo[1,2-*a*]pyrazine core.

**Figure 2:**
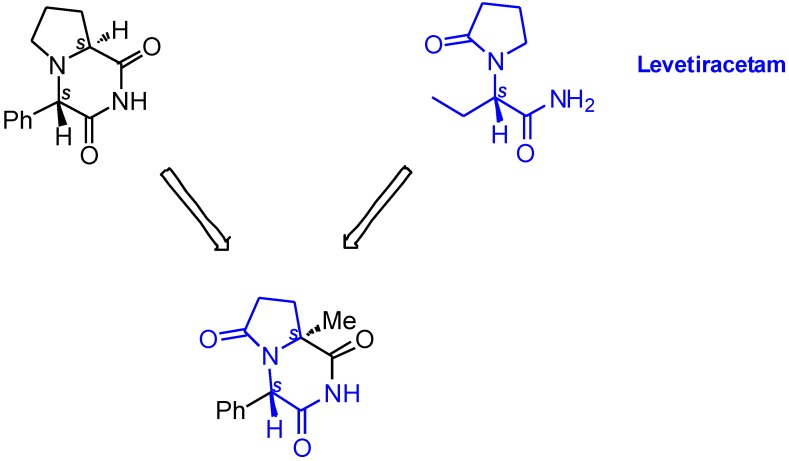
Hybrid compound of Levetiracetam and ADD408003.

To complement our previous extensive SAR investigations in this group of active compounds [7,8,9,10], we synthesized and pharmalogically evaluated a new series of ADD408003 derivatives. Since the mechanism of action of the parent molecule remains unknown, the new compounds have been designed according to diverse classical medicinal chemistry methods. First, we decided to examine if increasing the distance of the benzene ring from the imide moiety by methylene insertion would influence the anticonvulsant activity of ADD408003. Next, we asked whether replacing the hydrogen atom at C-4 of the parent compound with a non-epimerizable substituent would prevent the putative metabolic conversion to the inactive (4*R*,8a*S*) isomer, thus prolonging the anticonvulsant activity. Further, we incorporated alkyl residues in the C-4 position. Finally, based on the common structural motifs and the stereochemistry of ADD408003 and the potent broad-spectrum AED Levetiracetam, we have designed a hybrid of both molecules (Figure 2).

Since our previous studies on the stereochemistry-activity relationship of structurally related pyrido[1,2-*a*]pyrazines revealed interesting activity of (*R*,*R*) stereoisomers, we now focused on synthesizing both (*R*,*R*) and (*S*,*S*) enantiomers of the new ADD408003 derivatives [8].

## 2. Results and Discussion

### 2.1. Chemistry

**Scheme 1:**
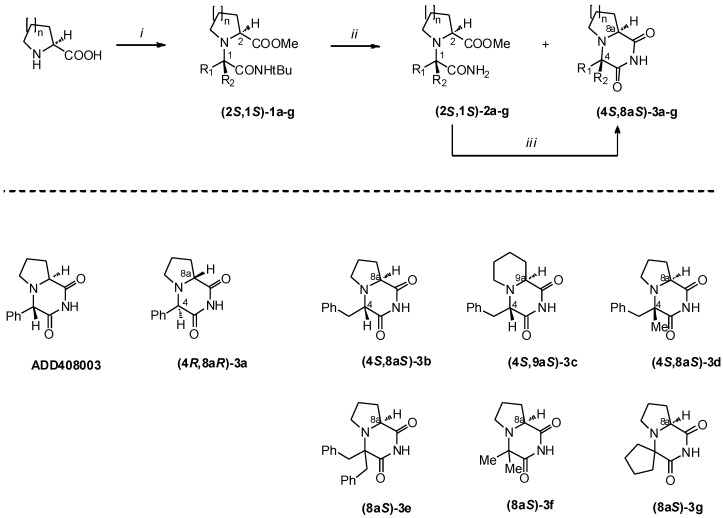
Synthesis of compounds **1–3a–g**.

Target compounds **3a–g** of the desired absolute configurations were obtained using the Ugi five-center four-component reaction (U-5C-4CR [11]) → amide N-de*tert*butylation → intramolecular cyclocondensation sequence [12], as shown on Scheme 1. For clarity, only the routes to **(4*S*,8a*S*)-3** and **(8a*S*)-3** isomers are shown and discussed. The respective opposite mirror images **(4*R*,8a*R*)-3** and **(8a*R*)-3** were synthesized by the same sequence, using (*R*)-amino acids as starting materials.

The appropriate (*S*)-amino acids were condensed with aldehydes or aliphatic ketones, *tert*-butyl isocyanide and methanol in the presence of catalytic amount of FeCl_3_ or TiCl_4_ to give Ugi adducts **1a–g** with chemical yields ranging from 17% to 64% (Table 1). When aldehydes or unsymmetrical ketones were employed as the starting materials, the reactions proceeded with the formation of the new stereocenters at carbons C-1 of the products **1a–d**. In cases of adducts **1a–c** the diastereoinduction was in favor of the **(2*S*,1*S*)** configurations, whereas equal amounts of **(2*S*,1*S*)-1d** and **(2*S*,1*R*)-1d** isomers were obtained for benzylmethyl ketone as the carbonyl component. In all cases the isomeric mixtures could be efficiently resolved by flash column chromatography on silica.

In the subsequent step, **(2*S*,1*S*)-1a–g** were subjected to *N*-de*tert*butylation mediated by BF_3_•2CH_3_COOH complex. Amido esters **(2*S*,1*S*)-2a–c** were obtained in good yields (43%–62%) and without loss of the stereochemical purity. Interestingly, in the reaction of Ugi adducts derived from ketones **(2*S*,1*S*)-1d** and **(2*S*)-1e–g**, the respective cyclized products **(4*S*,8a*S*)-3d**, **(8a*S*)-3f** and **(8a*S*)-3g** were formed in considerable (24%–70%) amounts. The amido ester **(2*S*)-2e** could not be isolated from the postreaction mixture due to the complete acid mediated deprotection/cyclisation of **(2*S*)-1e** to **(8a*S*)-3e**.

In the last step of the synthesis, amido esters **2** were converted to their cyclic derivatives **3** upon treatment with 1 eq. of NaOH in EtOH, with chemical yields ranging from 55% to 95%. The cyclocondensation of **(2*S*,1*S*)-2a–c** was accompanied by a slight degree of epimerization at carbon C-4. Pure diastereomers **(4*S*,8a*S*)-3a–c** were obtained by recrystallization.

*Reagents and conditions*: *i*. (a) carbonyl component, *t*BuNC, MeOH, FeCl_3(cat.)_ (or TiCl_4(cat.)_ for **1d–g**), MeOH, rt, 24 h (or 72 h for **1d–g**); (b) Chromatographic separation (**1a–d**); *ii*. BF_3_•2CH_3_COOH, 40–50 °C, 4–12 h; *iii*. NaOH, EtOH, rt, 10 min.

**Table 1:** Synthesis of compounds **1–3a–g**.

*U-5C-4CR*					*N-de-tert-butylation*		*Cyclocondensation*
Ugi adduct	Amino acid	Carbonyl component	Yield	Dr ^a^	Yield 1→2	Yield 1→3	Yield 1→3
**(2S,2S)-1a**	L-Proline	Benzaldehyde	64% ^b^	4:1	62%	- ^c^	65% ^d^
**(2S,2S)-1b**	L-Proline	Phenylacetaldehyde	54% ^b^	4:1	61%	-	69% ^d^
**(2S,2S)-1c**	L-Pipecoline	Phenylacetaldehyde	60% ^b^	4:1	43%	-	55% ^d^
**(2S,2S)-1d**	L-Proline	Phenylacetone	38% ^b^	1:1	46%	24%	88% ^d^
**(2S)-1e**	L-Proline	1,3-Diphenylacetone	17%	-	-	46%	-
**(2S)-1f**	L-Proline	Acetone	61%	-	33%	36%	95%
**(2S)-1g**	L-Proline	Cyclopentanone	60%	-	26%	70%	-

^a^ Dr of **(2*S*,1*S*)-1/(2*S*,1*R*)-1** astimated by ^1^H analyses of the crude postreaction mixtures (**1a–c**) or LC/MS (**1d**); ^b^ Sum of (2*S*,1*S*)-1 and **(2*S*,1*R*)-1** diastereoisomers; ^c^ Not applicable; ^d^ Yield of pure diastereoisomers.

It is well known that the U-5C-4CR reaction is highly versatile with regard to the substrate scope [11,12,13,14,15,16,17]. In this work and in our previous investigations, we have successfully coupled cyclic amino acids with aliphatic ketones [13]. However, to the best of our knowledge, aromatic ketones have not been employed as carbonyl components in this process. We were particularly interested in L-Proline and acetophenone adduct, as it could lead to the C-4 methylated ADD408003 derivative **(4*S*,8a*S*)-3h**. Propitiously, we observed by LC/MS that the U-5C-4CR product **1h** was formed after 1 day, at room temperature, with the use of a TiCl_4_ catalyst. The diastereoinduction slightly in favored **(2*S*,1*S*)-1h** (dr = 2:1, LC/MS). Unfortunately, attempts to optimize this particular reaction led to only a 13% yield after 7 days of stirring at room temperature.

**Scheme 2:**
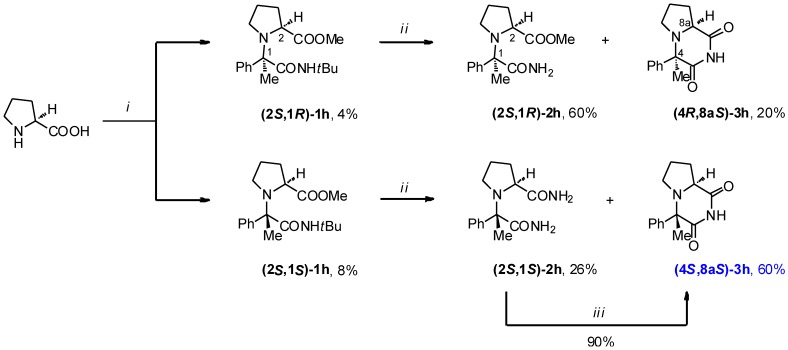
Synthesis of compounds **1–3h**.

**Figure 3:**
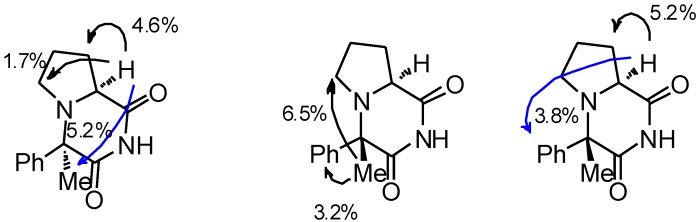
Selected nuclear Overhauser effect (nOe) correlations in **(4*R*,8a*S*)-3h** (left) and **(4*S*,8a*S*)-3h** (middle, right).

Nevertheless, enough **(4*S*,8a*S*)-3h** was obtained for initial evaluation in animal models of epilepsy, following the synthetic route described for **3a–g** (Scheme 2 and Figure 3). Similar to what had been observed for **1d–g** cyclic imides **3h** were formed upon treatment of the respective diastereomers of **1h** with BF_3_•2CH_3_COOH complex. Interestingly, cyclocondensation of **(2*S*,1*S*)-1h** diastereomer to **(4*S*,8a*S*)-3h** was more facile than in the case of its epimer **(2*S*,1*R*)-1h**.

*Reagents and conditions*: *i*. (a) PhCOCH_3_, *t*BuNC, MeOH, TiCl_4(cat.)_, MeOH, rt, 7 days; (b) Chromatographic separation; *ii*. BF_3_•2CH_3_COOH, 40–50 °C, 4–12 h; *iii*. NaOH, EtOH, rt, 10 min.

**Scheme 3:**
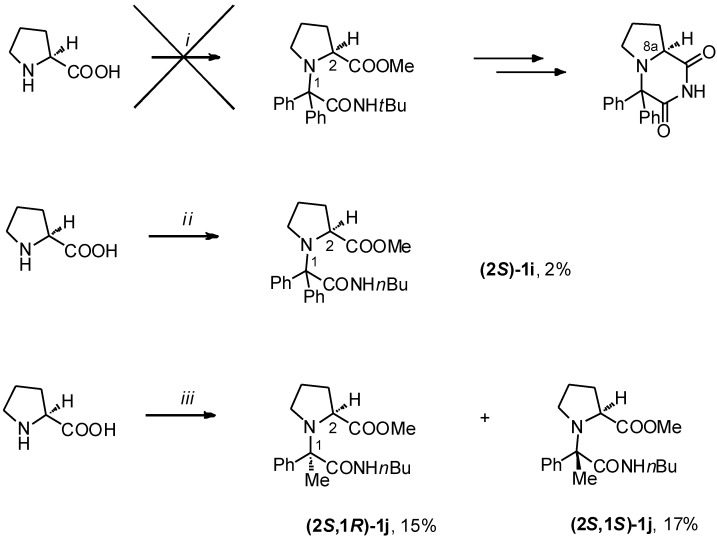
U-5C-4CR of L-proline aromatic ketones.

Encouraged by the results of U-5C-4CR of acetophenone, L-proline, *tert*-butyl isocyanide and MeOH, we tested if the biphenyl analog of ADD408003 could be obtained in the same manner as **3h** (Scheme 3). However, the more bulky benzophenone failed to react and no traces of Ugi adduct were detected by LC/MS of the crude postcondensation mixture. On the other hand, when *tert*-butyl isocyanide was replaced by the linear *n*-butyl isocyanide, we observed formation of the product **(2*S*)-1i** in a modest 2% yield. This was in agreement with our previous observations that steric factors played the most important role for the outcome of U-5C-4CR of ketones [13]. As a further example of this relationship, the yield of acetophenone adduct **1j** incresed from 13% to 32%, when less bulky *n*-butylisocyanide was used in lieu of the branched *tert*-butyl isomer. A loss of stereoselectivity was observed at the same time.

*Reagents and conditions*: *i*. Ph_2_CO, *t*BuNC, MeOH, TiCl_4(cat.)_, MeOH, rt, 7 days; *ii*. Ph_2_CO, *n*BuNC, MeOH, TiCl_4(cat.)_, MeOH, rt, 7 days; *iii*. (a) PhCOCH_3_, *n*BuNC, MeOH, TiCl_4(cat.)_, MeOH, rt, 7 days; (b) Chromatographic separation.

**Scheme 4:**
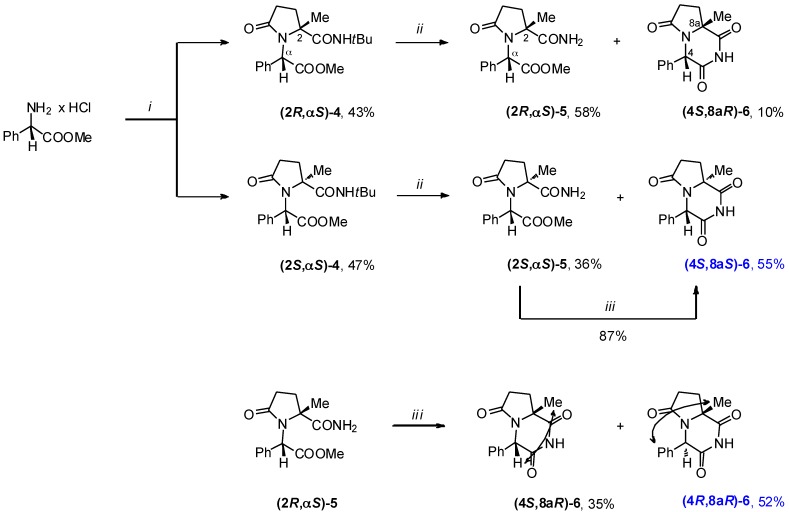
Synthesis of compounds **6**.

The desired **(4*R*,8a*R*)** and **(4*S*,8a*S*)** diastereomers of **6**, bearing oxygen atom at carbon C-6, were synthesized using the intramolecular Ugi five-center four-component reaction (U-4C-3CR) of methyl (*S*)-phenylglycinate, levulinic acid, *tert*-butyl isocyanide and MeOH as a key step of the synthetic sequence depicted in Scheme 4 [18,19,20]. The uncatalyzed reaction proceeded with a high yield and the resulting equimolar mixture of **(2*R*,α*S*)-4** and **(2*S*,α*S*)-4** was quantitatively separated by column chromatography on silica. The subsequent treatment of the respective diastereomers of **4** with BF_3_•2CH_3_COOH gave the dealkylated amido-esters **5** and cyclic imides **6**. Similar to what had been observed for dealkylation reactions of **1h**, acid-mediated cyclocondensation was more facile in case of the **(2*S*,α*S*)-4** diastereomer. Finally, base-mediated cyclization of **(2*R*,α*S*)-5** proceeded with a high degree of epimerization on C-4 of the products, resulting in the chromatographically separable mixture of **(4*S*,8a*R*)-6** and the desired **(4*R*,8a*R*)-6**. No epimerization took place in the analogous cyclocondensation reaction of **(2*S*,α*S*)-5**.

*Reagents and conditions*: *i*. (a) CH_3_COCH_2_CH_2_COOH, *t*BuNC, MeOH, MeOH, rt, 24 h; (b) Chromatographic separation; *ii*. BF_3_•2CH_3_COOH, 40–50 °C, 4–12 h; *iii*. NaOH, EtOH, rt, 10 min. The selected NOESY correlations have been shown for **(4*S*,8a*R*)-6** and **(4*R*,8a*R*)-6**.

### 2.2. Anticonvulsant Evaluation

Compounds 3 and 6 were evaluated in the *in vivo* animal models of epilepsy within the Anticonvulsant Screening Program (ASP) of The National Institute of Neurological Disorders and Stroke (NINDS), at The National Institutes of Health according to the established decision schemes and using protocols described in the Experimental Section of this article [21]. Primary anticonvulsant studies involved two tests: maximal electroshock seizure (MES) and subcutaneous Metrazol (scMET), in mice [22]. It is emphasized that nearly all clinically significant AEDs are protective in at least one of these two models, thus making them very useful tools for initial high througput screening of candidate anticonvulsants. The MES test employs an electrical stimulus to induce generalized tonic-clonic seizures and identifies which compounds prevent the spread of seizures. The scMET model utilizes chemically induced myoclonic seizures and recognizes the agents that act by raising the seizure threshold. Selected compounds were subjected to evaluation of anticonvulsant activity in the minimal clonic seizure (6 Hz) model of pharmacoresistant partial seizures [23,24,25]. Recently, it was found that Levetiracetam, a novel broad-spectrum AED, is ineffective in most of the classic tests (e.g., MES and scMET) and that it suppresses seizures in the 6 Hz model at the same time. Therefore, an expanded approach employed by the ASP is to utilize this test as a screening procedure to examine the compounds that are inactive in conventional models, but which can possess novel activity spectra. In addition to the primary anticonvulsant evaluation in the MES, scMET and 6Hz models, the acute neurological impairment (TOX) was assessed in *rotorod* test [26]. The results are summarized in Table 2 and Table 3.

**Table 2:** Anticonvulsant activity and neurotoxicity of compounds in the MES and scMET models following intraperitoneal (ip.) administration in mice.

Compound	Dose (mg/kg)		MES ^a^				scMET ^b^				TOX ^c^		
			0.5 h		4.0 h		0.5 h		4.0 h		0.5 h		4.0 h
**(4*R*,8a*R*)-3a**	100		0/3		0/3		0/1		0/1		0/8		0/4
	300		**1/1**		0/1		**1/1**		0/1		**4/4**		0/2
**(4*S*,8a*S*)-3b**	100		0/3		0/3		0/1		0/1		0/8		0/4
	300		**1/1**		0/1		0/1		0/1		0/4		0/2
**(4*R*,8a*R*)-3b**	100		0/3		0/3		0/1		0/1		0/8		0/4
	300		**1/1**		0/1		0/1		0/1		0/4		0/2
**(4*S*,9a*S*)-3c**	100		**1/3**		0/3		0/1		0/1		0/8		0/4
	300		**1/1**		0/1		**5/5**		0/1		04		0/2
**(4*R*,9a*R*)-3c**	100		**1/3**		0/3		0/1		0/1		0/8		0/4
	300		**1/1**		0/1		0/1		0/1		**4/4** ^d,e^		**2/2** ^d^
**(4*S*,8a*S*)-3d**	100		0/4		0/4		- ^f^		-		0/4		0/4
	300		-		-		-		-		-		-
**(4*R*,8a*R*)-3d**	100		0/4		0/4		-		-		0/4		0/4
	300		-		-		-		-		-		-
**(8a*S*)-3e**	100		0/4		0/4		0/4		0/4		0/4		0/4
	300		-		-		-		-		-		-
**(8a*R*)-3e**	100		0/4		0/4		0/4		0/4		0/4		0/4
	300		-		-		-		-		-		-
**(8a*S*)-3f**	100		0/1		0/1		0/1		0/1		**3/8**		**1/4** ^d^
	300		0/3		0/2		0/1		0/1		**4/4** ^g^		0/4
**(8a*S*)-3g**	100		0/4		0/4		0/4		0/4		0/4		0/4
	300		-		-		-		-		-		-
**(4*S*,8a*S*)-3h**	100		**3/4** ^h^		0/4		0/4		0/4		0/8		0/8
	300		-		-		-		-		-		-
**(4*S*,8a*S*)-6**	100		**3/4**		**3/4**		0/4		0/8		0/8		0/8
	300		-		-		-		-		-		-
**(4*R*,8a*R*)-6**	100		**3/4**		0/4		0/4		0/8		0/8		0/8
	300		-		-		-		-		-		-

^a^ Maximal electroshock test (number of animals protected/number of animals tested); ^b^ Subcutaneous Metrazole test (number of animals protected/number of animals tested); ^c^ Neurotoxicity test (number of animals exhibiting neurological toxicity/number of animals tested); ^d^ One animal died; ^e^ Unable to grasp rotorod; ^f^ Not determined; ^g^ Four animals died; ^h^ Active also in 1/4 at 0.25 h post administration.

**Table 3:** Anticonvulsant activity and neurotoxicity of compounds in the 6 Hz model following intraperitoneal (ip.) administration in mice.

Compound	Test ^a^	0.25 h	0.5 h	1.0 h	2.0 h	4.0 h
**(4*R*,8a*R*)-3a**	6 Hz ^b^	**4/4**	**4/4**	**1/4**	**1/4**	0/4
	TOX ^c^	-	-	-	-	-
**(4*S*,9a*S*)-3c**	6 Hz	**1/4**	**2/4**	0/4	0/4	0/4
	TOX	0/4	0/4	0/4	0/4	0/4
**(4*R*,9a*R*)-3c**	6 Hz	**3/4**	**1/4**	**1/4**	**1/4**	0/4
	TOX	0/4	0/4	0/4	0/4	0/4
**(4*S*,8a*S*)-3d**	6 Hz	**1/4**	**1/4**	0/4	0/4	0/4
	TOX	0/4	0/4	0/4	0/4	0/4
**(4*R*,8a*R*)-3d**	6 Hz	**3/4**	**2/4**	0/4	0/4	0/4
	TOX	0/4	0/4	0/4	0/4	0/4
**(8a*S*)-3e**	6 Hz	0/4	0/4	0/4	0/4	0/4
	TOX	0/4	0/4	0/4	0/4	0/4
**(8a*R*)-3e**	6 Hz	0/4	0/4	0/4	0/4	0/4
	TOX	0/4	0/4	0/4	0/4	0/4
**(8a*S*)-3f**	6 Hz	-	0/4	-	**1/4**	-
	TOX	-	**1/4**	-	0/4	-
**(8a*R*)-3f**	6 Hz ^d^	0/4	0/4	**1/3**	0/4	**1/4**
	TOX ^d^	**2/4** ^e^	**1/4** ^e^	**1/4** ^f^	0/4	0/4
**(8a*S*)-3g**	6 Hz	0/4	0/4	0/4	0/4	0/4
	TOX	0/4	0/4	0/4	0/4	0/4
**(8a*R*)-3g**	6 Hz ^d^	**1/2**	**2/2**	**2/2**	**3/3**	0/4
	TOX ^d^	**4/4** ^e,g,h,i^	**2/4** ^g^	**2/4** ^g^	**1/4** ^f^	0/4
**(4*S*,8a*S*)-3h**	6 Hz ^j^	**1/4**	**3/4**	0/4	0/4	0/4
	TOX ^j^	0/4	0/4	0/4	0/4	0/4
**(4*S*,8a*S*)-6**	6 Hz	**3/4**	**2/4**	**2/4**	0/4	0/4
	TOX	0/4	0/4	0/4	0/4	0/4

^a^ At dose 100 mg/kg; ^b^ 6 Hz test, 32 mA (number of animals protected/number of animals tested); ^c^ Neurotoxicity test (number of animals exhibiting neurological toxicity/number of animals tested); ^d^ At dose 50 mg/kg; ^e^ Clonic seizures; ^f^ One animal died; ^g^ Two animals died; ^h^ Unable to grasp rotorod; ^i^ Tremors; ^j^ At dose 75 mg/kg.

In contrast to the parent compound ADD408003, its opposite enantiomer **(4*R*,8a*R*)-3a** displayed only a weak activity in the ‘classical’ MES model (1/1 at 300 mg/kg, at 0.5 h). A considerable level of neurotoxicity was observed at the same dose and timepoint. An increase in the distance of the benzene ring from the imide moiety of ADD408003 and **(4*R*,8a*R*)-3a** by a methylene unit resulted in **(4*S*,8a*S*)-3b** and **(4*R*,8a*R*)-3b**, respectively. This structural modification did not improve the activity in MES model, when compared to parent molecules, although no signs of neurotoxicity were observed in the TOX test. The piperidine homologs of **(4*S*,8a*S*)-3b** and **(4*R*,8a*R*)-3b**, **(4*S*,9a*S*)-3c** and **(4*R*,9a*R*)-3c**, respectively, showed somewhat greater potency in the MES test (1/1 and 1/3 at 300 and 100 mg/kg, respectively, at 0.5 h). On the other hand, **(4*R*,9a*R*)-3c** isomer showed pronounced unwanted neurotoxic effect at 300 mg/kg. Replacing the hydrogen atom at C-4 with methyl group in **(4*S*,8a*S*)-3b** and **(4*R*,8a*R*)-3b** resulted in inactive **(4*S*,8a*S*)-3d** and **(4*R*,8a*R*)-3d**, respectively. Dibenzyl analogs **(8a*S*)-3e** and **(8a*R*)-3e** displayed no activity in MES model and no neurotoxicity in TOX test at a dose of 100 mg/kg. The alkyl derivatives **(8a*S*)-3f** and **(8a*S*)-3g** were devoid of desired anticonvulsant activity and the former produced severe neurotoxicity. The C-4 methylated analog of ADD408003, **(4*S*,8a*S*)-3h**, showed high levels of seizure protection in MES model (1/4 and 3/4 at 0.25 and 0.5 h, respectively, at 100 mg/kg) and lack of neurotoxic effects. However, the compound was not competitive when compared to the parent molecule, and due to the low synthetic accessibility it was not scheduled for secondary anticonvulsant evaluations. A comparable activity was observed for the *oxo-* derivatives **(4*S*,8a*S*)-6** and **(4*R*,8a*R*)-6**.

The anticonvulsant activity of investigated compounds in the scMET model in mice was markedly lower than in the MES screen. Only **(4*R*,8a*R*)-3a** and **(4*S*,9a*S*)-3c** showed considerable levels of seizure protection (1/1 and 5/5, respectively, at 0.5 h) at dose of 300 mg/kg.

To conclude the above results, the investigated compounds showed only moderate anticonvulsant activity in the ‘classical’ MES and scMET screening. Encouragingly, the levels of seizure protection in the 6 Hz model of pharmacoresistant epilepsy were markedly higher (Table 3).

Interestingly, the C-4 phenylmethyl derivatives **(4*R*,8a*R*)-3c** and **(4*R*,8a*R*)-3d** showed a slightly greater potency than their respective (4*S*,8a*S*) enantiomers in this test. Dibenzyl analogs **(8a*R*)-3e** and **(8a*S*)-3e** were devoid of anticonvulsant activity and neurotoxicity, whereas the alkyl derivatives **(8a*R*)-3f** and **(8a*S*)-3f** displayed severe motor impairment and proconvulsant effects.

Compounds **(4*R*,8a*R*)-3a** and **(4*S*,8a*S*)-6** were most potent in the primary 6 Hz screening, at both early and late time points. Therefore they were further subjected to *in vivo* quantification in this model (Table 4). They displayed median effective dose (ED_50_) values of 47.90 mg/kg and 126.19 mg/kg, respectively, at 0.25 h. Although the compounds proved less competitive than the parent ADD408003, their potencies and protective indices were comparable to those of standard AEDs.

**Table 4:** Quantification studies of **(4*R*,8a*R*)-3a** and **(4*S*,8a*S*)-6** in the 6 Hz and neurotoxicity tests following intraperitoneal (ip.) administration in mice.

Compound	ED_50_ 6 Hz (mg/kg)	TD_50_ (mg/kg) ^a^	PI ^b^ 6Hz	TPE ^c^ (h)
**(4*R*,8a*R*)-3a**	47.90 (35.6–71.08)	277.09 (245.78–350.13)	5.8	0.25
**(4*S*,8a*S*)-6**	126.19 (105.87–145.68)	262.22 (242.15–290.77)	2.1	0.25
**1** ^d^	9.93 (5.97–14.55)	121.5 (104.9–142.8)	12.2	0.25
**Phenytoin** ^e^	>60	41.0 (39.4–43.0)	-	-
**Phenobarbital** ^e^	14.8 (8.9–23.9)	69.0 (62.8–72.9)	4.7	0.5
**Ethosuximide** ^e^	167 (114–223)	341 (290–384)	2.0	0.25
**Levetiracetam** ^e^	19.4 (9.90–36.0)	>500	>25.8	1.0

Values in parentheses are 95% confidence intervals determined by probit analysis; ^a^ Neurotoxicity; ^b^ Protective index (TD_50_/ED_50_ 6 Hz); ^c^ Time to peak effect for the 6Hz test; ^d^ Data from Ref. [8]; ^e^ Data from Ref. [25]

## 3. Experimental Section

### 3.1. Chemistry

The NMR spectra were obtained on a Varian VNMRS 300 MHz (Agilent Technologies, Santa Clara, CA, USA) or Varian Inova 500 MHz (Agilent Technologies, Santa Clara, CA, USA) spectrometer at room temperature. Chemical shifts (*δ*) were expressed in parts per million (ppm) relative to tetramethylsilane (TMS) or residual solvent peaks used as the internal references. The following abbreviations were used to describe the signal patterns: s (singlet), d (doublet), t (triplet), q (quartet), m (multiplet), p (pseudo-) and b (broad-). Coupling constants (*J*) were in hertz (Hz). The FT-IR spectra (thin film on KBr pellets) were recorded on a FT-IR8300 instrument (Shimadzu Corporation, Kyoto, Japan). High resolution mass spectra (HRMS) were obtained using a LCT-TOF (Micromass) spectrometer (Waters Corporation, Milford, MA, USA) with electrospray ionization (ESI). Optical rotations were measured with a Perkin-Elmer 241 polarimeter (Perkin-Elmer, Waltham, MA, USA) using a sodium lamp (589 nm). Melting points were determined on an Electrothermal 9100 apparatus (Bibby-Scientific, Stone, UK) in open capillary tubes and were uncorrected. LC/MS analyses were performed using a Shimadzu Nexera UHPLC system (Shimadzu Corporation, Kyoto, Japan) with LCMS-2020 single quadrupole spectrometer (Shimadzu Corporation, Kyoto, Japan) equipped with an ESI ion source. Supelcosil LC-18-DB column (length: 25 mm, internal diameter: 4.6 mm, particle size: 5 μm, (Supelco, Bellefonte, PA, USA) was used. The analyte concentration was approximately 50 ng/mL, injection: 5 µL, flow rate 0.4 mL/min, temperature: 60 °C, mobile phase: acetonitrile/water 60:40 (v/v). Thin-layer chromatography (TLC) was run on Merck silica gel (60-F_254_) plates (Merck, Darmstadt, Germany). The spots were visualized by ultraviolet light (254 nm) or iodine vapors. Flash column chromatography (FC) was carried out on silica gel 60 (particle size: 0.040-0.063 mm, Merck, Darmstadt, Germany). Solvents were dried and purified by standard methods. Petroleum ether (PE) referred to the fraction boiling at 40–60 °C. All reagents were purchased from commercial sources and used as received. Compounds **(2*S,*1*S*)-1d-g** and **(2*S,*1*R*)-1d** were synthesized as previously described [13].

#### 3.1.1. Synthesis of Compounds **1** by U-5C-4CR Condensation

FeCl_3_ (for **1a–c**) or TiCl_4_ (for **1d–g**) (5 mol%) and isocyanide (1.0 eq.) were added to a stirred solution of appropriate α-amino acid (1.2 eq.) and carbonyl component (1.0 eq.) in MeOH (100 mL). The mixture was stirred at rt for 24 h (72 h for **1d–g**) and the volatiles were removed under reduced pressure. The resulting crude products were purified by FC.

*Methyl (2R,1R)-* and *(2R,1S)-1-(1-(tert-butylcarbamoyl)-1-phenylmethyl)-pyrrolidine-2-carboxylate* **(2R,1R)-1a** and **(2R,1S)-1a**. From D-proline (2.32 g, 20.16 mmol), benzaldehyde (1.78 mL, 16.80 mmol) and *tert*-butyl isocyanide (2.00 mL, 16.80 mmol); FC (PE/AcOEt 8:1 to 2:1): yield 3.43 g (64%): 2.26 g (42%) of **(2*R,*1*R*)-1a**, 0.45 g (8%) of **(2*R,*1*S*)-1a** and 0.72 g (14%) of diastereomeric mixture. **(2*R,*1*R*)-1a**: Yellow oil; TLC (PE/AcOEt 3:1): R_f_ = 0.15; [α]_D_ = +3.6 (*c* 0.546, CHCl_3_); IR (KBr): 759, 1171, 1215, 1362, 1454, 1520, 1673, 1736, 2842, 2967, 3295; ^1^H-NMR (CDCl_3_, 500 MHz): *δ* 1.41 (s, 9H, C(C*H*_3_)_3_), 1.78–1.92 (m, 3H, H-3, H-4, H′-4), 2.02 (m, 1H, H′-3), 2.70 (pq, ^2^*J* = ^3^*J*_1_ = ^3^*J*_2_ =7.5, 1H, H-5), 3.22 (m, 1H, H′-5), 3.30 (dd, ^3^*J*_1_ = 9.0, ^3^*J*_2_ = 4.0, 1H, H-2), 3.49 (s, 3H, OC*H*_3_), 4.09 (s, 1H, H-α), 7.23–7.32 (m, 5H, H-2′, H-3′, H-4′, H-5′, H-6′), 7.70 (bs, 1H, CON*H*); ^13^C-NMR (CDCl_3_, 125 MHz): *δ* 24.3 (C-4), 28.8 (C(*C*H_3_)_3_), 30.8 (C-3), 50.7 (*C*(CH_3_)_3_), 51.6 (O*C*H_3_), 54.4 (C-5), 61.6 (C-2), 74.0 (C-α), 128.2 (C-4′), 128.5 (C-2′, C-6′), 129.2 (C-3′, C-5′), 137.3 (C-1′), 171.0 (*C*ONH), 175.9 (*C*OOCH_3_); HRMS (ESI+) calcd for C_18_H_26_N_2_O_3_Na 341.1841 (M+Na)^+^ found 341.1845. **(2*R,*1*S*)-1a**: Yellow oil; TLC (PE/AcOEt 3:1): R_f_ = 0.26; [α]_D_ = +81.8 (*c* 0.672, CHCl_3_); IR (KBr): 758, 1164, 1203, 1363, 1453, 1513, 1678, 1739, 2856, 2964, 3317; ^1^H-NMR (CDCl_3_, 500 MHz): *δ* 1.35 (s, 9H, C(C*H*_3_)_3_), 1.77 (m, 1H, H-4), 1.83 (m, 1H, H′-4), 1.91 (m, 1H, H-3), 2.10 (m, 1H, H′-3), 2.53 (m, ^2^*J* = 9.0, ^3^*J*_1_=^3^*J*_2_ = 7.5, 1H, H-5), 2.92 (4d, ^2^*J* = 9.0, ^3^*J*_1_ = 7.5, ^3^*J*_2_ = 4.5, 1H, H′-5), 3.55 (dd, ^3^*J*_1_ = 9.4, ^3^*J*_2_ = 3.0, 1H, H-2), 3.69 (s, 3H, OC*H*_3_), 4.20 (s, 1H, H-α), 7.26-7.35 (m, 6H, H-2′, H-3′, H-4′, H-5′, H-6′, CON*H*); ^13^C-NMR (CDCl_3_, 125 MHz): *δ* 23.9 (C-4), 28.6 (C(*C*H_3_)_3_), 29.8 (C-3), 50.7 (*C*(CH_3_)_3_), 50.9 (C-5), 51.8 (O*C*H_3_), 63.7 (C-2), 73.2 (C-α), 128.0 (C-4′), 128.3 (C-2′, C-6′), 129.0 (C-3′, C-5′), 136.9 (C-1′), 170.6 (*C*ONH), 175.3 (*C*OOCH_3_); HRMS (ESI+) calcd for C_18_H_26_N_2_O_3_Na 341.1841 (M+Na)^+^ found 341.1850.

*Methyl (2S,1S)-* and *(2S,1R)-1-(1-(tert-butylcarbamoyl)-3-phenyl-1-ethyl)-pyrrolidine-2-carboxylate* **(2*S,*1*S*)-1b** and **(2*S,*1*R*)-1b**. From L-proline (2.32 g, 20.16 mmol), phenylacetaldehyde (2.10 mL, 16.80 mmol) and *tert*-butyl isocyanide (2.00 mL, 16.80 mmol); FC (PE/AcOEt 7:1 to 2:1): yield 3.02 g (54%): 2.01 g (36%) of **(2*S,*1*S*)-1b**, 0.39 g (7%) of **(2*S,*1*R*)-1b** and 0.62 (11%) of diastereomeric mixture. **(2*S,*1*S*)-1b**: Yellow oil; TLC (PE/AcOEt 3:1): R_f_ = 0.25; [α]_D_ = −26.2 (*c* 1, CHCl_3_); IR (KBr): 745, 1092, 1173, 1204, 1362, 1454, 1520, 1670, 1736, 2874, 2966, 3310; ^1^H-NMR (CDCl_3_, 500 MHz): *δ* 1.26 (s, 9H, C(C*H*_3_)_3_), 1.76 (m, 1H, H-4), 1.81 (m, 1H, H′-4), 1.89 (m, 1H, H-3), 1.98 (m, 1H, H′-3), 2.88 (m, ^2^*J* = 14.0, ^3^*J* = 6.5, 1H, C*H*_2_), 2.91 (m, 1H, H-5), 3.18 (m, 1H, H′-5), 3.23 (dd, ^2^*J* = 14.0, ^3^*J* = 8.0, 1H, C*H*_2_), 3.48 (t, ^3^*J* = 7.5, 1H, H-2), 3.66 (m, 1H, H-1), 3.70 (s, 3H, OC*H*_3_), 6.52 (bs, 1H, CON*H*), 7.16–7.30 (m, 5H, H-2′, H-3′, H-4′, H-5′, H-6′); ^13^C-NMR (CDCl_3_, 125 MHz): *δ* 23.6 (C-4), 28.6 (C(*C*H_3_)_3_), 30.7 (C-3), 36.5 (*C*H_2_), 50.7 (C-5), 51.9 (O*C*H_3_), 51.9 (*C*(CH_3_)_3_), 60.5 (C-2), 67.3 (C-1), 126.3 (C-4′), 128.4 (C-3′, C-5′), 129.0 (C-2′, C-6′), 138.8 (C-1′), 171.5 (*C*ONH), 175.9 (*C*OOCH_3_); HRMS (ESI+) calcd for C_19_H_28_N_2_O_3_Na: 355.1998 (M+Na)^+^ found 355.2004. **(2*S,*1*R*)-1b**: White wax; M.p. 75–77 °C; TLC (PE/AcOEt 3:1): R_f_ = 0.31; [α]_D_ = +9.0 (*c* 1, CHCl_3_); IR (KBr): 748, 1207, 1454, 1520, 1678, 1732, 2874, 2962, 3317; ^1^H-NMR (CDCl_3_, 500 MHz): *δ* 1.33 (s, 9H, C(C*H*_3_)_3_), 1.72–1.81 (m, 4H, H-3, H′-3, H-4, H′-4), 2.89 (dd, ^2^*J* = 14.5, ^3^*J* = 9.5, 1H, C*H*_2_), 2.91 (m, 1H, H-5), 2.96 (m, 1H, H′-5), 3.25 (m, 1H, H-2), 3.38 (dd, ^2^*J* = 14.5, ^3^*J* = 5.0, 1H, C*H*_2_), 3.62 (dd, ^3^*J*_1_ = 9.5, ^3^*J*_2_ = 5.0, 1H, H-1), 3.66 (s, 3H, OC*H*_3_), 7.16-7.29 (m, 5H, H-2′, H-3′, H-4′, H-5′, H-6′), 7.58 (bs, 1H, CON*H*); HRMS (ESI+) calcd for C_19_H_28_N_2_O_3_Na: 355.1998 (M+Na)^+^ found 355.1995.

*Methyl (2R,1R)-* and *(2R,1S)-1-(1-(tert-butylcarbamoyl)-3-phenyl-1-ethyl)-pyrrolidine-2-carboxylate* **(2*R,*1*R*)-1b** and **(2*R,*1*S*)-1b**. From D-proline (2.32 g, 20.16 mmol), phenylacetaldehyde (2.10 mL, 16.80 mmol) and *tert*-butyl isocyanide (2.00 mL, 16.80 mmol); FC (PE/AcOEt 7:1 to 2:1): yield 2.53 g (45%): 1.25 g (22%) of **(2*R,*1*R*)-1b**, 0.87 g (16%) of **(2*R,*1*S*)-1b** and 0.42 (7%) of diastereomeric mixture. **(2*R,*1*R*)-1b**: Yellow oil; [α]_D_ = +33.1 (*c* 1.3, CHCl_3_); HRMS (ESI+) calcd for C_19_H_28_N_2_O_3_Na: 355.1998 (M+Na)^+^ found 355.2008. **(2*R,*1*S*)-1b**: White wax; M.p. 65–70 °C; [α]_D_ = −6.9 (*c* 1, CHCl_3_); HRMS (ESI+) calcd for C_19_H_28_N_2_O_3_Na: 355.1998 (M+Na)^+^ found 355.1999.

*Methyl (2S,1S)-* and *(2S,1R)-1-(1-(tert-butylcarbamoyl)-3-phenyl-1-ethyl)-piperidine-2-carboxylate* **(2*S,*1*S*)-1c** and **(2*S,*1*R*)-1c**. From L*-*pipecolinic acid (2.60 g, 20.16 mmol), phenylacetaldehyde (2.10 mL, 16.80 mmol) and *tert*-butyl isocyanide (2.00 mL, 16.80 mmol); FC (PE/AcOEt 9:1 to 2:1): yield 3.49 g (60%): 2.32 g (40%) of **(2*S,*1*S*)-1c**, 0.83 (14%) of **(2*S,*1*R*)-1c** and 0.34 g (6%) of diastereomeric mixture. **(2*S,*1*S*)-1c**: Colorless oil; TLC (PE/AcOEt 3:1): R_f_ = 0.40; [α]_D_ = −33.6 (*c* 1, CHCl_3_); IR (KBr): 748, 1161, 1366, 1454, 1512, 1678, 1732, 2858, 2936; ^1^H-NMR (CDCl_3_, 500 MHz): *δ* 1.25 (s, 9H, C(C*H*_3_)_3_), 1.43 (m, 1H, H-4), 1.58 (m, 3H, H′-4, H-5, H′-5), 1.76 (m, 1H, H-3), 1.83 (m, 1H, H′-3), 2.61 (m, ^2^*J* = 12.5, ^3^*J* = 5.0, 1H, H-6), 2.88 (dd, ^2^*J* = 13.0, ^3^*J* = 4.0, 1H, H-2), 3.13–3.22 (m, 2H, H′-6, C*H*_2_), 3.24 (dd, ^2^*J* = 8.5, ^3^*J* = 4.0, 1H, C*H*_2_′), 3.44 (dd, ^3^*J_1_* = 7.5, ^3^*J_2_* = 4.0, 1H, H-1), 3.68 (s, 3H, OC*H*_3_), 5.95 (bs, 1H, CON*H*), 7.15–7.25 (m, 5H, H-2′, H-3′, H-4′, H-5′, H-6′); ^13^C-NMR (CDCl_3_, 125 MHz): *δ* 22.2 (C-4), 25.8 (C-3), 28.6 (C(*C*H_3_)_3_), 29.8 (C-5), 36.2 (*C*H_2_), 45.9 (C-6), 51.0 (*C*(CH_3_)_3_), 51.7 (O*C*H_3_), 62.7 (C-2), 69.1 (C-1), 126.1 (C-4′), 128.2 (C-2′, C-6′), 129.3 (C-3′, C-5′), 139.3 (C-1′), 169.9 (*C*ONH), 174.3 (*C*OOCH_3_); HRMS (ESI+) calcd for C_20_H_30_N_2_O_3_Na 369.2154 (M+Na)^+^ found 369.2158. **(2*S,*1*R*)-1c**: Colorless oil; TLC (PE/AcOEt 3:1): R_f_ = 0.50; [α]_D_ = −0.6 (*c* 1, CHCl_3_); IR (KBr): 740, 799, 1196, 1225, 1454, 1516, 1682, 1744, 2858, 2963, 3344; ^1^H-NMR (CDCl_3_, 500 MHz): *δ* 1.34 (s, 9H, C(C*H*_3_)_3_), 1.44 (m, 2H, H-4, H-5), 1.58 (m, 1H, H′-5), 1.70 (m, 2H, H′-4, H-6), 2.53 (m, ^2^*J* = 11.5, ^3^*J*_1_ = 10.0, ^3^*J*_2_ = 2.5, 1H, H′-3), 2.76 (m, 1H, H-C*H*_2_), 2.79 (m, 1H, H′-6), 3.14 (dd, ^3^*J*_1_ = 9.5, ^3^*J*_2_ = 4.0, 1H, H-2), 3.28 (dd, 1H, ^2^*J* = 14.5, ^3^*J*_2_ = 6.0, C*H*_2_′), 3.48 (dd, ^3^*J*_1_ = 12.0, ^3^*J*_2_ = 6.0, 1H, H-1), 3.63 (s, 3H, OC*H*_3_), 7.18 (tt, ^3^*J* = 7.0, ^4^*J* = 2.0, 1H, H-4′), 7.23 (tt, ^3^*J* = 7.0, ^4^*J* = 2.0, 2H, H-3′, H-5′), 7.27 (d, ^3^*J* = 7.0, 2H, H-2′, H-6′), 7.40 (bs, 1H, CON*H*); HRMS (ESI+) calcd for C_20_H_30_N_2_O_3_Na 369.2154 (M+Na)^+^ found 369.2161.

*Methyl (2R,1R)-* and *(2R,1S)-1-(1-(tert-butylcarbamoyl)-3-phenyl-1-ethyl)-piperidine-2-carboxylate* **(2*R,*1*R*)-1c** and **(2*R,*1*S*)-1c**. From D*-*pipecolinic acid (2.60 g, 20.16 mmol), phenylacetaldehyde (2.10 mL, 16.80 mmol) and *tert*-butyl isocyanide (2.00 mL, 16.80 mmol); FC (PE/AcOEt 9:1 to 2:1): yield 3.20 g (55%): 2.11 g (36%) of **(2*R,*1*R*)-1c**, and 1.09 (19%) of **(2*R,*1*S*)-1c**. **(2*R,*1*R*)-1c**: Colorless oil; [α]_D_ = +31.2 (*c* 0.720, CHCl_3_); HRMS (ESI+) calcd for C_20_H_30_N_2_O_3_Na 369.2154 (M+Na)^+^ found 369.2155. **(2*S,*1*R*)-1c**: Colorless oil; [α]_D_ = +1.9 (*c* 0.880, CHCl_3_); HRMS (ESI+) calcd for C_20_H_30_N_2_O_3_Na 369.2154 (M+Na)^+^ found 369.2159.

*Methyl (2R,1S)-* and *(2R,1R)-1-(1-(tert-butylcarbamoyl)-1-methyl-3-phenyl-1-ethyl)-pyrrolidine-2-carboxylate* **(2*R*,1*S*)-1d** and **(2*R*,1*R*)-1d**. From D*-*proline (2.32 g, 20.16 mmol), phenyl-2-propanone (2.24 mL, 16.80 mmol) and *tert-*butyl isocyanide (2.00 mL, 16.80 mmol); FC (PE/AcOEt 12:1 to 7:1): yield 1.80 g (31%): 0.76 g (13%) of **(2*R*,1*S*)-1d**, 0.58 g (10%) of **(2*R*,1*R*)-1d** and 0.46 g (8%) of diastereomeric mixture. **(2*R*,1*S*)-1d**: Pale-yellow wax; M.p. 61–68 °C; TLC (PE/AcOEt 5:1): R_f_ = 0.37; [α]_D_ = +30.1 (*c* 0.811, CHCl_3_); LC/MS: 347 [M+H]^+^, retention time: 17.2 min; HRMS (ESI+) calcd for C_20_H_30_N_2_O_3_Na: 369.2154 (M+Na)^+^ found 369.2157. **(2*R*,1*R*)-1d**: Pale-yellow wax; M.p. 35-42 °C; TLC (PE/AcOEt 5:1): R_f_ = 0.26; [α]_D_ = +9.1 (*c* 0.920, CHCl_3_); LC/MS: 347 [M+H]^+^, retention time: 20.0 min; HRMS (ESI+) calcd for C_20_H_30_N_2_O_3_Na: 369.2154 (M+Na)^+^ found 369.2162.

*Methyl (2R)-1-(1-(tert-butylcarbamoyl)-1-benzyl-3-phenyl-1-ethyl)-pyrrolidine-2-carboxylate* **(2*R*)-1e**. From L-proline (2.32 g, 20.16 mmol), diphenyl-2-propanone (3.54 g, 16.80 mmol) and *tert-*butyl isocyanide (2.00 mL, 16.80 mmol); FC (PE/AcOEt 12:1 to 8:1): yield 1.63 g (23%). Yellow oil; TLC (PE/AcOEt 3:1): R_f_ = 0.35; [α]_D_ = +38.0 (*c* 1, CHCl_3_); HRMS (ESI+) calcd for C_26_H_34_N_2_O_3_Na: 445.2467 (M+Na)^+^ found 445.2474.

*Methyl (2R)-1-(1-(tert-butylcarbamoyl)-1-methyl-1-ethyl)-pyrrolidine-2-carboxylate* **(2*R*)-1f**. From L-proline (2.32 g, 20.16 mmol), 2-propanone (1.24 mL, 16.80 mmol) and *tert-*butyl isocyanide (2.00 mL, 16.80 mmol); FC (PE/AcOEt 9:1 to 3:1): yield 2.04 g (45%). White wax; M.p. 40–43 °C; TLC (PE/AcOEt 3:1): R_f_ = 0.35; [α]_D_ = +32.5 (*c* 0.950, CHCl_3_); HRMS (ESI+) calcd for C_14_H_26_N_2_O_3_Na: 293.1841 (M+Na)^+^ found 293.1848.

*Methyl (2R)-1-(1-(tert-butylcarbamoyl)-cyclopentyl)-pyrrolidine-2-carboxylate* **(2*R*)-1g**. From L-proline (2.32 g, 20.16 mmol), cyclopentanone (1.48 mL, 16.80 mmol) and *tert-*butyl isocyanide (2.00 mL, 16.80 mmol); FC (gradient: PE/AcOEt 10:1 to 3:1): yield 2.74 g (55%). Colorless oil; TLC (PE/AcOEt 5:1): R_f_ = 0.41; [α]_D_ = +15.2 (*c* 0.513, CHCl_3_); HRMS (ESI+) calcd for C_16_H_28_N_2_O_3_Na: 319.1998 (M+Na)^+^ found 319.2004.

*Methyl (2S,1S)-* and *(2S,1R)-1-(1-(tert-butylcarbamoyl)-1-methyl-1-phenylmethyl)-pyrrolidine-2-carboxylate* **(2*S*,1*S*)-1h** and **(2*S*,1*R*)-1h**. From L*-*proline (2.32 g, 20.16 mmol), acetophenone (2.02 mL, 16.80 mmol) and *tert-*butyl isocyanide (2.00 mL, 16.80 mmol); FC (PE/AcOEt 10:1 to 5:1): yield 0.72 g (14%): 0.40 g (8%) of **(2*S*,1*S*)-1h**, 0.20 (4%) of **(2*S*,1*R*)-1h** and 0.12 g (2%) of diastereomeric mixture. **(2*S*,1*S*)-1h**: Pale-yellow powder; M.p. 109–111 °C; TLC (PE/EA 5:1): R_f_ = 0.23; [α]_D_ = −43.3 (*c* 0.800; CHCl_3_); IR (KBr): 760, 1169, 1203, 1447, 1502, 1679, 1738, 2872, 2963, 3300; ^1^H-NMR (CDCl_3_, 500 MHz): *δ* 1.40 (s, 9H, C(C*H*_3_)_3_), 1.68 (s, 3H, C*H*_3_), 1.71 (m, 1H, H-4), 1.77 (m, 1H, H′-4), 1.87 (m, 2H, H-3, H′-3), 2.93 (pq, ^2^*J* = ^3^*J*_1_ = ^3^*J*_2_ =8.5, 1H, H-5), 3.11 (m, ^2^*J* = 9.5, ^3^*J*_1_ = 6.0, ^3^*J*_2_ = 4.0, 1H, H′-5), 3.35 (dd, ^3^*J*_1_ = 8.5, ^3^*J*_2_ = 3.0, 1H, H-2), 3.43 (s, 3H, OC*H*_3_), 7.22 (t, ^3^*J* = 7.5, 1H, H-4′), 7.28 (t, ^3^*J* = 7.5, 2H, H-3′, H-5′), 7.43 (d, ^3^*J* = 7.5, 2H, H-2′, H-6′), 7.91 (bs, 1H, CON*H*); ^13^C-NMR (CDCl_3_, 125 MHz): *δ* 18.1 (*C*H_3_), 25.4 (C-4), 28.9 (C(*C*H_3_)_3_), 31.9 (C-3), 49.6 (C-5), 50.9 (*C*(CH_3_)_3_), 51.7 (O*C*H_3_), 61.2 (C-2), 69.1 (C-1), 127.7 (C-2′, C-6′), 127.8 (C-4′), 128.3 (C-3′, C-5′), 141.5 (C-1′), 173.4 (*C*ONH), 177.0 (*C*OOCH_3_); LC/MS: 333 [M+H]^+^, retention time: 14.0 min; HRMS (ESI+) calcd for C_19_H_28_N_2_O_3_Na: 355.1998 (M+Na)^+^ found 355.1996. **(2*S*,1*R*)-1h**: White powder; M.p. 112–113 °C; TLC (PE/EA 5:1): R_f_ = 0.32; [α]_D_ = −14.7 (*c* 0.600; CHCl_3_); IR (KBr): 758, 1167, 1203, 1447, 1503, 1679, 1741, 2871, 2964, 3351; ^1^H-NMR (CDCl_3_, 500 MHz): *δ* 1.21 (s, 9H, C(C*H*_3_)_3_), 1.62 (s, 3H, C*H*_3_), 1.64–1.78 (m, 2H, H-4, H′-4), 1.89–2.00 (m, 2H, H-3, H′-3), 2.37 (m, ^2^*J* = ^3^*J*_1_ = 9.5, ^3^*J*_2_ = 7.0, 1H, H-5), 2.99 (m, ^2^*J* = 9.5, ^3^*J*_1_ = 7.0, ^3^*J*_2_ = 3.0, 1H, H′-5), 3.73 (s, 3H, OC*H*_3_), 3.79 (dd, ^3^*J*_1_ = 9.0, ^3^*J*_2_ = 2.5, 1H, H-2), 7.22 (t, ^3^*J* = 7.5, 1H, H-4′), 7.31 (t, ^3^*J* = 7.5, 2H, H-3′, H-5′), 7.42 (bs, 1H, CON*H*), 7.55 (d, ^3^*J* = 7.5, 2H, H-2′, H-6′); ^13^C-NMR (CDCl_3_, 125 MHz): *δ* 16.4 (*C*H_3_), 24.9 (C-4), 28.6 (C(*C*H_3_)_3_), 32.2 (C-3), 50.0 (C-5), 50.8 (*C*(CH_3_)_3_), 52.2 (O*C*H_3_), 61.7 (C-2), 70.5 (C-1), 127.3 (C-4′), 127.5 (C-2′, C-6′), 128.2 (C-3′, C-5′), 142.2 (C-1′), 173.4 (*C*ONH), 177.2 (*C*OOCH_3_); LC/MS: 333 [M+H]^+^, retention time: 17.0 min; HRMS (ESI+) calcd for C_19_H_28_N_2_O_3_Na: 355.1998 (M+Na)^+^ found 355.1985.

#### 3.1.2. Synthesis of Compounds **2** and **5** by BF_3_•2CH_3_COOH Mediated *N*-de*tert*butylation

The appropriate Ugi product **1** or **4** was dissolved in BF_3_•2CH_3_COOH (~36% BF_3_ basis, 3 mL per 1 mmol of substrate), and stirred at 40–50 °C until total consumption of the starting material (TLC), typically for 4–12 h. The resulting solution was cooled, poured onto excess of crushed ice and made alkaline with 25% aqueous solution of ammonia. The mixture was extracted with DCM (3 × 50 mL). The combined organic phase was washed with water (30 mL), brine (30 mL), dried over anhydrous MgSO_4_, filtered and concentrated under reduced pressure. The residue was purified by FC.

*Methyl (2R,1R)-1-(1-carbamoyl-1-phenylmethyl)-pyrrolidine-2-carboxylate* **(2*R,*1*R*)-2a**. From **(2*R*,α*R*)-1a** (2.13 g, 6.70 mmol); FC (PE/AcOEt 1:1 to 1:2); Yield 1.21 g (69%). White powder; M.p. 155–157 °C; TLC (AcOEt): R_f_ = 0.28; [α]_D_ = −4.0 (*c* 0.612, CHCl_3_); HRMS (ESI+) calcd for C_18_H_26_N_2_O_3_Na 258.1210 (M+Na)^+^ found 258.1208.

*Methyl (2S,1S)-1-(1-carbamoyl-3-phenyl-1-ethyl)-pyrrolidine-2-carboxylate* **(2*S*,1*S*)-2b**. From **(2*S*,α*S*)-1b** (1.91 g, 5.75 mmol); FC (PE/AcOEt 2:1 to 0:1, then EA/MeOH 95:5); Yield 1.02 g (61%). Yellow oil; TLC (AcOEt): R_f_ = 0.36; [α]_D_ = −35.5 (*c* 0.827, CHCl_3_); IR (KBr): 758, 1173, 1213, 1456, 1678, 1735, 2854, 2925, 2951, 3025, 3192; ^1^H-NMR (CDCl_3_, 500 MHz): *δ* 1.66–1.86 (m, 1H, H-3, H-4, H′-4), 1.93 (m, 1H, H′-3), 2.84 (m, 1H, H-5), 2.93 (dd, ^2^*J* = 14.5, ^3^*J* = 7.5, 1H, C*H*_2_), 3.23 (m, 2H, H′-5, C*H*_2_), 3.66 (t, ^3^*J* = 7.5, 1H, H-2), 3.70–3.72 (m, 4H, H-1, OC*H*_3_), 5.40 (bs, 1H, CON*H*), 6.97 (bs, 1H, CON*H′*), 7.20 (t, ^3^*J* = 7.5, 1H, H-4′), 7.23 (d, ^3^*J* = 7.5, 2H, H-2′, H-6′), 7.28 (t, ^3^*J* = 7.5, 2H, H-3′, H-5′); ^13^C-NMR (CDCl_3_, 125 MHz): *δ* 24.0 (C-4), 30.9 (C-3), 36.9 (*C*H_2_), 52.3 (O*C*H_3_), 52.7 (C-5), 60.8 (C-2), 66.6 (C-1), 126.7 (C-4′), 128.8 (C-3′, C-5′), 129.1 (C-2′, C-6′), 138.7 (C-1′), 175.8 (*C*ONH), 176.4 (*C*OOCH_3_); HRMS (ESI+) calcd for C_15_H_20_N_2_O_3_Na: 299.1372 (M+Na)^+^ found 299.1401.

*Methyl (2R,1R)-1-(1-carbamoyl-3-phenyl-1-ethyl)-pyrrolidine-2-carboxylate* **(2*R*,1*R*)-2b**. From **(2*R*,1*R*)-1b** (1.20 g, 3.61 mmol); FC (PE/AcOEt 2:1 to 0:1, then EA/MeOH 95:5); Yield 0.51 g (52%). Yellow oil; [α]_D_ = +40.8 (*c* 1.1; CHCl_3_); HRMS (ESI+) calcd for C_15_H_20_N_2_O_3_Na: 299.1372 (M+Na)^+^ found 299.1380.

*Methyl (2S,1S)-1-(1-carbamoyl-3-phenyl-1-ethyl)-piperidine-2-carboxylate* **(2*S,*1*S*)-2c**. From **(2*S*,1*S*)-1c** (2.79 g, 8.06 mmol); FC (PE/AcOEt 3:1 to 1:1); Yield 1.00 g (43%). White wax; M.p. 80–84 °C; TLC (AcOEt): R_f_ = 0.55; [α]_D_ = −47.1 (*c* 0.670, CHCl_3_); IR (KBr): 698, 748, 1173, 1200, 1439, 1686, 1736, 2854, 2936, 3209, 3445; ^1^H-NMR (CDCl_3_, 500 MHz): *δ* 1.42 (m, 1H, H-4), 1.59 (m, 3H, H′-4, H-5, H′-5), 1.76 (m, 1H, H-3), 1.84 (m, 1H, H-6), 2.55 (m, 1H, H′-3), 2.94 (dd, ^2^*J* = 13.5, ^3^*J* = 5.5, 1H, H-β), 3.16 (m, 1H, H′-6), 3.19 (dd, ^2^*J* = 13.5, ^3^*J* = 8.5, 1H, H′-β), 3.41 (dd, ^3^*J*_1_ = 8.5, ^3^*J*_2_ = 5.5, 1H, H-α), 3.48 (dd, ^3^*J*_1_ = 7.0, ^3^*J*_2_ = 4.0, 1H, H-2), 3.68 (s, 3H, OC*H*_3_), 5.77 (bs, 1H, CON*H*), 6.24 (bs, 1H, CON*H′*), 7.18 (tt, ^3^*J* = 7.0, ^4^*J* = 1.5, 1H, H-4′), 7.23 (tt, ^3^*J* = 6.0, ^4^*J* = 1.5, 2H, H-3′, H-5′), 7.26 (tt, ^3^*J* = 6.0, ^4^*J* = 1.5, 2H, H-2′, H-6′); ^13^C-NMR (CDCl_3_, 125 MHz): *δ* 22.0 (C-4), 25.7 (C-3), 29.6 (C-5), 35.8 (C- β), 46.1 (C-6), 51.8 (O*C*H_3_), 62.7 (C-2), 68.2 (C-α), 126.2 (C-4′), 128.3 (C-3′, C-5′), 129.1 (C-2′, C-6′), 173.4 (*C*OOCH_3_), 174.2 (*C*ONH_2_); HRMS (ESI+) calcd for C_16_H_22_N_2_O_3_Na 313.1528 (M+Na)^+^ found 313.1522.

*Methyl (2R,1R)-1-(1-carbamoyl-3-phenyl-1-ethyl)-piperidine-2-carboxylate* **(2*R,*1*R*)-2c**. From **(2*R*,1*R*)-1c** (1.63 g, 4.71 mmol); FC (PE/AcOEt 3:1 to 1:1); Yield 0.74 g (54%). Pale-yellow wax; M.p. 79–81 °C; [α]_D_ = −45.5 (*c* 0.540, CHCl_3_); HRMS (ESI+) calcd for C_16_H_22_N_2_O_3_Na 313.1528 (M+Na)^+^ found 313.1531.

*Methyl (2S,1S)-1-(1-carbamoyl-1-methyl-3-phenyl-1-ethyl)-pyrrolidine-2-carboxylate* **(2*S*,1*S*)-2d** and *(4S,8aS)-4-Methyl-4-benzylperhydropyrrolo[1,2-a]pyrazine-1,3-dione* **(4*S*,8a*S*)-3d**. From **(2*S*,1*S*)-1d** (1.54 g, 4.45 mmol); FC (PE/AcOEt 4:1 to 0:1): Yield 70%: 0.60 g (46%) of **(2*S*,1*S*)-2d** and 0.28 g (24%) of **(4*S*,8a*S*)-3d**. **(2*S*,1*S*)-2d**: White powder, M.p. 121–123 °C; TLC (AcOEt): R_f_ = 0.54; [α]_D_ = −12.3 (*c* 0.790, CHCl_3_); IR (KBr): 702, 1171, 1202, 1456, 1506, 1684, 1736, 2843, 2870, 2950, 3445; ^1^H-NMR (CDCl_3_, 500 MHz): *δ* 1.12 (s, 3H, C*H*_3_), 1.76 (m, 1H, H-4), 1.81–1.95 (m, 2H, H-3, H′-4), 2.12 (m, 1H, H′-3), 2.70 (d, ^2^*J* = 12.5, 1H, C*H*_2_), 2.79 (m, ^2^*J* = ^3^*J*_1_ = 9.0,^ 3^*J*_2_ = 6.0, 1H, H-5), 2.99-3.08 (m, 2H, H′-5, C*H*_2_), 3.72 (s, 3H, OC*H*_3_), 3.84 (dd, ^3^*J*_1_ = 9.5, ^3^*J*_2_ = 3.5, 1H, H-2), 7.16–7.28 (m, 5H, H-Ar), 7.34 (bs, 1H, CON*H*); ^13^C-NMR (CDCl_3_, 75 MHz): *δ* 14.3 (*C*H_3_), 24.7 (C-4), 32.0 (C-3), 45.0 (*C*H_2_), 48.9 (C-5), 52.1 (O*C*H_3_), 58.9 (C-2), 66.5 (C-1), 126.8 (C-4′), 128.0 (C-2′, C-6′), 130.2 (C-3′, C-5′), 136.4 (C-1′), 177.2 (*C*ONH), 177.5 (*C*OOCH_3_); HRMS (ESI+) calcd for C_16_H_22_N_2_O_3_Na 313.1528 (M+Na)^+^ found 313.1525. **(4*S*,8a*S*)-3d**: White powder; M.p. 151–153 °C; TLC (PE/AcOEt 3:1): R_f_ = 0.20; [α]_D_ = −20.6 (*c* 0.775; CHCl_3_); IR (KBr): 702, 770, 1194, 1265, 1508, 1701, 2819, 2953, 2988, 3196; ^1^H-NMR (CDCl_3_, 500 MHz): *δ* 1.36 (s, 1H, C*H*_3_), 1.86 (m, 2H, H-7, H-8), 2.36 (m, 2H, H′-7, H′-8), 2.58 (q, ^2^*J* = ^3^*J*_1_ = ^3^*J*_2_ = 8.5, 1H, H-6), 3.01 (d, ^2^*J* = 13.5, 1H, C*H*_2_), 3.11 (m, 1H, H′-6), 3.17 (d,^ 2^*J* = 13.5, 1H, C*H*_2_’), 4.20 (dd, ^3^*J*_1_ = 8.5, ^3^*J*_2_ = 3.5, 1H, H-8a), 7.20 (m, 2H, H-Ar), 7.33 (m, 3H, H-Ar), 8.00 (bs, 1H, N*H*); ^13^C-NMR (CDCl_3_, 125 MHz): *δ* 20.9 (*C*H_3_), 22.2 (C-7), 27.5 (C-8), 43.0 (*C*H_2_), 47.7 (C-6), 59.1 (C-8a), 63.0 (C-4), 127.1 (C-4′), 128.2 (C-2′, C-6′), 130.4 (C-3′, C-5′), 135.3 (C-1′), 174.5 (C-3), 175.0 (C-1); HRMS (ESI+) calcd for C_15_H_18_N_2_O_2_Na: 281.1266 (M+Na)^+^ found 281.1272.

*Methyl (2R,1R)-1-(1-carbamoyl-1-methyl-3-phenyl-1-ethyl)-pyrrolidine-2-carboxylate* **(2*R*,1*R*)-2d** and *(4R,8aR)-4-Methyl-4-benzylperhydropyrrolo[1,2-a]pyrazine-1,3-dione* **(4*R*,8a*R*)-3d**. From **(2*R*,1*R*)-1d** (1.31 g, 3.79 mmol); FC (PE/AcOEt 4:1 to 0:1); Yield 65%: 0.48 g (44%) of **(2*R*,1*R*)-2d** and 0.21 g (21%) of **(4*R*,8a*R*)-3d**. **(2*R*,1*R*)-2d**: Pale yellow, M.p. 118–120 °C; [α]_D_ = +11.7 (*c* 0.880, CHCl_3_); HRMS (ESI+) calcd for C_16_H_22_N_2_O_3_Na 313.1528 (M+Na)^+^ found 313.1524. **(4*R*,8a*R*)-3d**: Pale-yellow powder; M.p. 150–152 °C; [α]_D_ = +20.9 (*c* 0.942; CHCl_3_); HRMS (ESI+) calcd for C_15_H_18_N_2_O_2_Na: 281.1266 (M+Na)^+^ found 281.1267.

*(8aS)-4,4-Dibenzylperhydropyrrolo[1,2-a]pyrazine-1,3-dione* **(8a*S*)-3e**. From **(2*S*)-1e** (1.12 g, 2.65 mmol); FC (PE/AcOEt 10:1 to 3:1), then recrystallization from PE/Et_2_O; Yield 0.41 g (46%). White powder, M.p. 121–122 °C; TLC (PE/AcOEt 3:1): R_f_ = 0.36; [α]_D_ = −8.3 (*c* 0.775, CHCl_3_); IR (KBr): 700, 748, 1218, 1267, 1354, 1452, 1504, 1697, 2831, 2926, 3028, 3207; ^1^H-NMR (500 MHz, CDCl_3_): *δ* 1.76-1.92 (m, 3H, H-7, H′-7, H-8), 1.99 (m, 1H, H-8, H′-8), 3.11 (d, ^2^*J* = 15.0, 1H, C*H*_2_), 3.16–3.27 (m, 4H, H-6, H′-6, C*H*_2_, C*H*_2_′), 3.30 (t, ^3^*J* = 7.0, 1H, H-8a), 3.35 (d, ^2^*J* = 14.0, 1H, C*H*_2_′), 7.12–7.31 (m, 10H, H-Ar), 7.77 (bs, 1H, N*H*), ^13^C-NMR (CDCl_3_, 125 MHz): *δ* 21.7 (C-7), 26.3 (C-8), 41.0, 41.2 (*C*H_2_, *C*H_2_′), 45.2 (C-6), 58.8 (C-8a), 68.3 (C-4), 126.6, 127.3, 128.1, 128.6, 130.06, 130.11, 136.0, 136.5 (C-Ar), 172.1 (C-3), 174.9 (C-1); HRMS (ESI+) calcd for C_21_H_22_N_2_O_2_Na: 357.1579 (M+Na)^+^ found 357.1577.

*(8aR)-4,4-Dibenzylperhydropyrrolo[1,2-a]pyrazine-1,3-dione* **(8a*R*)-3e**. From **(2*R*)-1e** (1.30 g, 3.08 mmol); FC (PE/AcOEt 10:1 to 3:1), then recrystallization from PE/Et_2_O; Yield: 0.35 g (34%). White powder, M.p. 116–118 °C; [α]_D_ = +7.7 (*c* 0.803, CHCl_3_); HRMS (ESI+) calcd for C_21_H_22_N_2_O_2_Na: 357.1579 (M+Na)^+^ found 357.1589.

*Methyl (2S)-1-(1-carbamoyl-1-methyl-1-ethyl)-pyrrolidine-2-carboxylate* **(2*S*)-2f** and *(8aS)-4,4-Dimethylperhydropyrrolo[1,2-a]pyrazine-1,3-dione* **(8a*S*)-3f**. From **(2*S*)**-**1f** (1.27 g, 4.70 mmol); FC (PE/AcOEt 5:1 to 0:1, then AcOEt/MeOH 95/5); Yield 69%: 0.33 g (33%) of **(2*S*)-2f** and 0.31 g (36%) of **(8a*S*)-3f**. **(2*S*)-2f**: White powder; M.p. 56–58 °C; TLC (AcOEt): R_f_ = 0.37; [α]_D_ = −38.4 (*c* 0.906, CHCl_3_); IR (KBr): 1203, 1435, 1578, 1681, 1736, 2874, 2951, 3206; ^1^H-NMR (CDCl_3_, 500 MHz): *δ* 1.24 (s, 3H, C*H*_3_), 1.26 (s, 3H, C*H*_3_’), 1.74–1.92 (m, 3H, H-3, H-4, H′-4), 1.89 (m, 1H, H′-3), 2.04 (m, 1H, H′-3), 2.72 (td, ^2^*J* = ^3^*J*_1_ = 9.0, ^3^*J*_2_ = 6.0, 1H, H-5), 3.02 (m, ^2^*J* = 8.5, ^3^*J*_1_ = 6.5, ^3^*J*_2_ = 3.0, 1H, H′-5), 3.61 (dd, ^3^*J*_1_ = 9.5,^ 3^*J*_2_ = 3.0, 1H, H-2), 3.71 (s, 3H, OC*H*_3_), 5.39 (bs, 1H, CON*H*), 7.44 (bs, 1H, CON*H′*); ^13^C-NMR (CDCl_3_, 125 MHz): *δ* 20.0 (*C*H_3_), 23.5 (*C*H_3_’), 24.8 (C-4), 31.7 (C-3), 48.4 (C-5), 52.0 (O*C*H_3_), 59.4 (C-2), 62.2 (C-α), 177.4 (*C*OOCH_3_), 179.8 (*C*ONH_2_); HRMS (ESI+) calcd for C_10_H_18_N_2_O_3_Na: 237.1215 (M+Na)^+^ found 237.1218. **(8a*S*)-3f**: White powder, M.p. 105–107 °C; TLC (PE/AcOEt 3:1): R_f_ = 0.10; [α]_D_ = +15.1 (*c* 0.933, CHCl_3_); IR (KBr): 1157, 1261, 1315, 1454, 1701, 2820, 2920, 2982, 3163; ^1^H-NMR (500 MHz, CDCl_3_): *δ* 1.43 (s, 3H, C*H*_3_), 1.46 (s, 3H, C*H*_3_’), 1.86 (m, 2H, H-7, H′-7), 2.26 (m, 2H, H-8, H′-8), 2.56 (pq, ^2^*J* = ^3^*J*_1_ = 7.5, ^3^*J*_2_ = 8.5, 1H, H-6), 3.01 (m, ^2^*J* = 8.5, ^3^*J*_1_ = 7.0, ^3^*J*_2_ = 5.5, 1H, H′-6), 3.89 (dd, ^3^*J*_1_ = 8.0, ^3^*J*_2_ = 4.0, 1H, H-8a), 7.78 (bs, 1H, N*H*), ^13^C-NMR (CDCl_3_, 125 MHz): *δ* 22.2, 23.8, 24.0 (C-7, 2×*C*H_3_), 27.5 (C-8), 47.7 (C-6), 59.6 (C-4), 60.0 (C-8a), 174.3 (C-3), 176.9 (C-1).

*Methyl (2R)-1-(1-carbamoyl-1-methyl-1-ethyl)-pyrrolidine-2-carboxylate* **(2*R*)-1f** and *(8aR)-4,4-Dimethylperhydropyrrolo[1,2-a]pyrazine-1,3-dione* **(8a*R*)-3f**. From **(2*R*)-1f** (2.66 g, 9.85 mmol); FC (PE/AcOEt 5:1 to 0:1, then AcOEt/MeOH 95/5); Yield 75%: 0.31 g (14%) of **(2*R*)-2f** and 1.13 g (61%) of **(8a*R*)-3f**. **(2*R*)-1f**: Pale-yellow powder; M.p. 53–56 °C; [α]_D_ = +36.1 (*c* 1.085, CHCl_3_); HRMS (ESI+) calcd for C_10_H_18_N_2_O_3_Na: 237.1215 (M+Na)^+^ found 237.1231. **(8a*R*)-3f**: White powder, M.p. 110–112 °C; [α]_D_ = +14.2 (*c* 0.847, CHCl_3_).

*Methyl (2S)-1-(1-carbamoyl-1-cyclopentyl)-pyrrolidine-2-carboxylate* **(2*S*)-2g** and *(8a’S)-perhydrospiro[cyclopentane-1,4'-pyrrolo[1,2-a]pyrazine]-1′,3′-dione* **(8a*S*)-3g**. From **(2*S*)-1g** (0.74 g, 2.50 mmol); FC (PE/AcOEt 3:1 to 0:1, then AcOEt/MeOH 95/5); Yield 96%: 0.16 g (26%) of **(2*S*)-2g** and 0.36 g (70%) of **(8a*S*)-3g**. **(2*S*)-2g**: Brown oil; TLC (AcOEt): R_f_ = 0.43; [α]_D_ = +3.8 (*c* 0.987, CHCl_3_); IR (KBr): 1200, 1454, 1569, 1680, 1732, 2872, 2953, 3441; ^1^H-NMR (CDCl_3_, 500 MHz): *δ* 1.56–1.67 (m, 4H), 1.68–2.11 (m, 8H), 2.24 (m, 1H, H′-3), 2.67 (m, ^2^*J* = ^3^*J*_1_ = 9.0, ^3^*J*_2_ = 6.5, 1H, H-5), 3.05 (m, ^2^*J* = 8.5, ^3^*J*_1_ = 6.0, ^3^*J*_2_ = 2.5, 1H, H′-5), 3.65 (dd, ^3^*J*_1_ = 9.0,^ 3^*J*_2_ = 3.0, 1H, H-2), 3.72 (s, 3H, OC*H*_3_), 5.40 (bs, 1H, CON*H*), 7.56 (bs, 1H, CON*H′*); ^13^C-NMR (CDCl_3_, 125 MHz): *δ* 24.7, 25.0, 25.2, 31.3, 31.7, 35.9, 49.3 (C-5), 52.0 (O*C*H_3_), 60.0 (C-2), 72.9 (C-1), 177.5 (*C*OOCH_3_), 179.9 (*C*ONH_2_); HRMS (ESI+) calcd for C_12_H_20_N_2_O_3_Na: 263.1372 (M+Na)^+^ found 263.1386. **(8a*S*)-3g**: White powder, M.p. 84–85 °C; TLC (PE/AcOEt 3:1): R_f_ = 0.11; [α]_D_ = +41.0 (*c* 0.550, CHCl_3_); IR (KBr): 1176, 1246, 1327, 1456, 1697, 2814, 2874, 2959, 3234; ^1^H-NMR (500 MHz, CDCl_3_): *δ* 1.69–197 (m, 8H, H-cPent), 2.05 (m, 1H, H-7′), 2.22 (m, 1H, H-8′), 2.34 (m, 2H, H′-7′, H′-8′), 2.45 (q, ^2^*J* = ^3^*J*_1_ = ^3^*J*_2_ = 8.5, 1H, H-6′), 3.07 (m, ^2^*J* = 8.5 = ^3^*J*_1_ = 8.5, ^3^*J*_2_ = 4.0, 1H, H′-6′), 3.89 (dd, ^3^*J*_1_ = 8.5, ^3^*J*_2_ = 2.5, 1H, H-8a′), 7.90 (bs, 1H, N*H*), ^13^C-NMR (CDCl_3_, 125 MHz): *δ* 22.2 (C-7′), 24.6, 25.1 (2×C-cPent), 27.4 (C-8′), 33.8, 37.4 (2×C-cPent), 48.2 (C-6′), 60.6 (C-8a′), 69.6 (C-4′), 174.1 (C-3′), 176.3 (C-1′); HRMS (ESI-) calcd for C_11_H_15_N_2_O_2_: 207.1134 (M−H)^−^ found 207.1130.

*Methyl (2R)-1-(1-carbamoyl-1-cyclopentyl)-pyrrolidine-2-carboxylate* **(2*R*)-2g** and *(8a’S)-perhydrospiro[cyclopentane-1,4′-pyrrolo[1,2-a]pyrazine]-1′,3′-dione* **(8a*R*)-3g**. From **(2*R*)-1g** (1.02 g, 3.45 mmol); FC (PE/AcOEt 3:1 to 0:1, then AcOEt/MeOH 95/5); Yield 81%: 0.25 g (30%) of **(2*R*)-2g** and 0.37 g (51%) of **(8a*R*)-3g**. **(2*R*)-2g**: Brown oil; [α]_D_ = −4.0 (*c* 0.833, CHCl_3_); HRMS (ESI+) calcd for C_12_H_20_N_2_O_3_Na: 263.1372 (M+Na)^+^ found 263.1371. **(8a*R*)-3g**: Pale-yellow powder, M.p. 85–87 °C; [α]_D_ = −43.8 (*c* 0.807, CHCl_3_); HRMS (ESI-) calcd for C_11_H_15_N_2_O_2_: 207.1134 (M−H)^−^ found 207.1127.

*Methyl (2S,1S)-1-(1-carbamoyl-1-methyl-1-phenylmethyl)-pyrrolidine-2-carboxylate* **(2*S*,1*S*)-2h** and *(4S,8aS)-4-Methyl-4-phenyl-perhydropyrrolo[1,2-a]pyrazine-1,3-dione* **(4*S*,8a*S*)-3h**. From **(2*S*,1*S*)-1h** (200 mg, 0.60 mmol); FC (PE/AcOEt 6:1 to 0:1); Yield 86%: 43 mg (26%) of **(2*S*,1*S*)-2h** and 88 mg (60%) of **(4*S*,8a*S*)-3h**. **(2*S*,1*S*)-2h**: White powder; M.p. 152–154 °C; TLC (AcOEt): R_f_ = 0.35; [α]_D_ = +50.1 (*c* 0.600; CHCl_3_); IR (KBr): 695, 754, 1169, 1205, 1376, 1681, 1737, 2842, 2923, 3179, 3413; ^1^H-NMR (CDCl_3_, 500 MHz): *δ* 1.72 (s, 3H, C*H*_3_), 1.78 (m, 2H, H-4, H′-4), 1.87 (m, 2H, H-3, H′-3), 2.97 (m, 1H, H-5), 3.20 (m, ^2^*J* = 9.5, ^3^*J*_1_ = 6.5, ^3^*J*_2_ = 4.0, 1H, H′-5), 3.39 (dd, ^3^*J*_1_ = 8.5, ^3^*J*_2_ = 3.5, 1H, H-2), 3.44 (s, 3H, OC*H*_3_), 5.71 (bs, 1H, CON*H*), 7.24 (t, ^3^*J* = 7.5, 1H, H-4′), 7.30 (t, ^3^*J* = 7.5, 2H, H-3′, H-5′), 7.49 (d, ^3^*J* = 7.5, 2H, H-2′, H-6′), 7.72 (bs, 1H, CON*H′*); ^13^C-NMR (CDCl_3_, 125 MHz): *δ* 18.5 (*C*H_3_), 25.4 (C-4), 31.8 (C-3), 49.8 (C-5), 51.9 (O*C*H_3_), 61.4 (C-2), 69.2 (C-α), 127.8 (C-2′,C-6′), 128.1 (C-4′), 128.4 (C-3′, C-5′), 141.5 (C-1′), 177.2 (*C*ONH) 177.4 (*C*OOCH_3_); HRMS (ESI+) calcd for C_15_H_20_N_2_O_3_Na: 299.1372 (M+Na)^+^ found 299.1362. **(4*S*,8a*S*)-3h**: White powder; M.p. 72–73 °C; TLC (PE/AcOEt 3:1): R_f_ = 0.38; [α]_D_ = −166.1 (*c* 0.800; CHCl_3_); IR (KBr): 702, 764, 1249, 1693, 2814, 2987, 3221; ^1^H-NMR (CDCl_3_, 500 MHz): *δ* 1.65 (s, 3H, C*H*_3_), 1.84–2.00 (m, 2H, H-7, H′-7), 2.13–2.24 (m, 1H, H-8), 2.24–2.32 (m, 1H, H′-8), 2.62 (q, ^2^*J* = ^3^*J*_1_ = ^3^*J*_2_ = 8.5, 1H, H-6), 3.30 (td, ^2^*J* = ^3^*J*_1_ = 8.5, ^3^*J*_2_ = 3.0, 1H, H′-6), 3.65 (dd, ^3^*J*_1_ = 8.0, ^3^*J*_2_ = 2.5, 1H, H-8a), 7.29 (t, ^3^*J* = 7.0, 1H, H-4′), 7.35 (t, ^3^*J* = 7.0, 2H, H-3′, H-5′), 7.46 (d, ^3^*J* = 7.0, 2H, H-2′, H-6′), 8.31 (bs, 1H, N*H*); ^13^C-NMR (CDCl_3_, 125 MHz): *δ* 22.8 (*C*H_3_), 26.6 (C-7), 27.6 (C-8), 47.4 (C-6), 60.2 (C-8a), 66.5 (C-4), 125.7 (C-2′, C-6′), 128.4 (C-4′), 129.2 (C-3′, C-5′), 141.3 (C-1′), 174.5 (C-3), 175.0 (C-1); HRMS (ESI+) calcd for C_14_H_16_N_2_O_2_Na: 267.1110 (M+Na)^+^ found 267.1118.

*Methyl (2S,1R)-1-(1-carbamoyl-1-methyl-1-phenylmethyl)-pyrrolidine-2-carboxylate* **(2*S*,1*R*)-2h** and *(4R,8aS)-4-Methyl-4-phenyl-perhydropyrrolo[1,2-a]pyrazine-1,3-dione* **(4*R*,8a*S*)-3h**. From **(2*S*,1*R*)-1h** (200 mg, 0.60 mmol); FC (PE/AcOEt 6:1 to 0:1); Yield 80%: 98 mg (60%) of **(2*S*,1*R*)-2h** and 28 mg (20%) of **(4*R*,8a*S*)-3h**. **(2*S*,1*R*)-2h**: White powder; M.p. 124–125 °C; TLC (AcOEt): R_f_ = 0.55; [α]_D_ = −43.6 (*c* 0.800; CHCl_3_); IR (KBr): 700, 1169, 1208, 1446, 1683, 1734, 2872, 2950, 3421; ^1^H-NMR (CDCl_3_, 500 MHz): *δ* 1.62–1.68 (m, 5H, H-4, H′-4, C*H*_3_), 1.96 (m, 2H, H-3, H′-3), 2.38 (m, ^2^*J* = ^3^*J*_1_ = 9.5, ^3^*J*_2_ = 6.5, 1H, H-5), 3.02 (m, ^2^*J* = 9.5, ^3^*J*_1_ = 7.0, ^3^*J*_2_ = 3.0, 1H, H′-5), 3.71 (s, 3H, OC*H*_3_), 3.84 (dd, ^3^*J*_1_ = 8.5, ^3^*J*_2_ = 3.5, 1H, H-2), 5.41 (bs, 1H, CON*H*), 7.25 (t, ^3^*J* = 7.5, 1H, H-4′), 7.33 (t, ^3^*J* = 7.5, 2H, H-3′, H-5′), 7.51 (bs, 1H, CON*H′*), 7.56 (d, ^3^*J* = 7.5, 2H, H-2′, H-6′); ^13^C-NMR (CDCl_3_, 125 MHz): *δ* 18.4 (*C*H_3_), 24.9 (C-4), 32.0 (C-3), 50.0 (C-5), 52.4 (O*C*H_3_), 61.3 (C-2), 69.8 (C-α), 127.6 (C-4′), 127.8 (C-2′, C-6′), 128.4 (C-3′, C-5′), 141.2 (C-1′), 177.3 (*C*ONH), 177.4 (*C*OOCH_3_); HRMS (ESI+) calcd for C_15_H_20_N_2_O_3_Na: 299.1372 (M+Na)^+^ found 299.1360. **(4*R*,8a*S*)-3h**: White powder; M.p. 126–128 °C; TLC (PE/AcOEt 3:1): R_f_ = 0.22; [α]_D_ = −80.0 (*c* 0.700; CHCl_3_); IR (KBr): 701, 758, 1234, 1705, 2830, 2977, 3217; ^1^H-NMR (CDCl_3_, 500 MHz): *δ* 1.70-1.90 (m, 5H, H-7, C*H*_3_), 2.22 (m, 2H, H-8), 2.40 (q, ^2^*J* = ^3^*J*_1_ = ^3^*J*_2_ = 8.0, 1H, H-6), 2.49 (td, ^2^*J* = ^3^*J*_1_ = 8.0, ^3^*J*_2_ = 4.0, 1H, H′-6), 3.67 (t, ^3^*J* = 8.0, 1H, H-8a), 7.31 (t, ^3^*J* = 7.5, 1H, H-4′), 7.37 (t, ^3^*J* = 7.5, 2H, H-3′, H-5), 7.56 (d, ^3^*J* = 7.5, 2H, H-2′, H-6′), 7.96 (bs, 1H, N*H*); ^13^C-NMR (CDCl_3_, 125 MHz): *δ* 14.2 (*C*H_3_), 21.4 (C-7), 25.8 (C-8), 46.2 (C-6), 58.7 (C-8a), 65.7 (C-4), 127.4 (C-2′, C-6′), 128.3 (C-4′), 128.7 (C-3′, C-5′), 141.1 (C-1′), 172.5 (C-3), 176.1 (C-1); HRMS (ESI+) calcd for C_14_H_16_N_2_O_2_Na: 267.1110 (M+Na)^+^ found 267.1106.

Methyl (2S,αS)-α-(2-carbamoyl-2-methyl-5-oxopyrrolidin-1-yl)-α-phenylacetate **(2S,αS)-5** and (4S,8aS)-4-phenyl-8a-methylperhydropyrrolo[1,2-a]pyrazine-1,3,6-trione **(4*S*,8a*S*)-6**. From **(2*S*,α*S*)-4** (2.65 g, 7.66 mmol); FC (PE/AcOEt 3:1 to 0:1, then AcOEt/MeOH 95/5); Yield 91%: 0.81 g (36%) of **(2*S*,α*S*)-5** and 1.08 g (55%) of **(4*S*,8a*S*)-6**. **(2*S*,α*S*)-5**: White powder; M.p. 174–175 °C; TLC (AcOEt): R_f_ = 0.29; [α]_D_ = +18.5 (*c* 0.700; CHCl_3_); IR (KBr): 723, 757, 1209, 1399, 1605, 1679, 1742, 3007, 3193, 3351; ^1^H-NMR (CDCl_3_, 500 MHz): *δ* 1.14 (s, 3H, C*H*_3_), 2.06 (m, 1H, H-3), 2.37-2.40 (m, 2H, H′-3, H-4), 2.55 (1H, H′-4), 3.75 (s, 3H, OC*H*_3_), 5.20 (s, 1H, H-α), 5.39 (bs, 1H, CON*H*), 7.24 (bs, 1H, CON*H′*), 7.36-7.40 (m, 3H, H-3′, H-4′, H-5′), 7.42-7.46 (m, 2H, H-2′, H-6′); ^13^C-NMR (CDCl_3_, 125 MHz): *δ* 23.3 (C*H*_3_), 28.6 (C-4), 35.4 (C-3), 53.2 (OC*H*_3_), 61.5 (C-2), 68.0 (C-α), 125.2 (C-2′, C-6′), 129.4 (C-4′), 133.0 (C-1′), 171.5 (C-5), 176.4 (*C*ONH_2_), 176.5 (*C*OOCH_3_); HRMS (ESI+) calcd for C_15_H_18_N_2_O_4_Na: 313.1164 (M+Na)^+^ found 313.1172. **(4*S*,8a*S*)-6**: White powder; M.p. 164–166 °C; TLC (AcOEt): R_f_ = 0.60; [α]_D_ = +50.4 (*c* 0.800; CHCl_3_); IR (KBr): 758, 1188, 1290, 1341, 1710, 2839, 3104, 3193; ^1^H-NMR (CDCl_3_, 500 MHz): *δ* 1.16 (s, 3H, C*H*_3_), 2.17 (m, 1H, H-8), 2.48-2.58 (m, 2H, H-7, H′-8), 2.66 (m, 1H, H′-7), 6.29 (s, 1H. H-4), 7.32-7.40 (m, 5H, H-Ar), 8.54 (bs, 1H, N*H*); ^13^C-NMR (CDCl_3_, 125 MHz): *δ* 25.1 (C*H*_3_), 28.8 (C-8), 30.6 (C-7), 55.5 (C-8a), 62.4 (C-4), 127.0 (C-2′, C-6′), 128.6 (C-4′), 129.1 (C-3′, C-5′), 135.2 (C-1′), 167.8 (C-6), 173.8 (C-1), 174.1 (C-3); HRMS (ESI-) calcd for C_14_H_13_N_2_O_3_: 257.0926 (M−H)^−^ found 257.0916.

*Methyl (2R,αS)-α-(2-carbamoyl-2-methyl-5-oxopyrrolidin-1-yl)-α-phenylacetate* **(2*R*,α*S*)-5** and *(4S,8aS)-4-phenyl-8a-methylperhydropyrrolo[1,2-a]pyrazine-1,3,6-trione* **(4*S*,8a*R*)-6**. From **(2*R*,α*S*)-4** (1.46 g, 4.22 mmol); FC (PE/AcOEt 3:1 to 0:1, then AcOEt/MeOH 95/5); Yield 65%: 0.71 g (58%) of **(2R,αS)-5** and 0.10 g (10%) of **(4*S*,8a*R*)-6**. **(2*R*,α*S*)-5**: White powder; M.p. 174–175 °C; TLC (AcOEt): R_f_ = 0.25; [α]_D_ = –124.5 (*c* 0.6; CHCl_3_); IR (KBr): 721, 754, 1268, 1401, 1605, 1692, 3007, 3193, 3369; ^1^H-NMR (CDCl_3_, 500 MHz): *δ* 1.33 (s, 3H, C*H*_3_), 2.00 (m, 1H, H-3), 2.42-2.61 (m, 2H, H′-3, H-4, H′-4), 2.55 (1H, H′-4), 3.79 (s, 3H, OC*H*_3_), 4.77 (s, 1H, H-α), 5.72 (bs, 1H, CON*H*), 7.32–7.40 (m, 5H, H-Ar), 8.48 (bs, 1H, CON*H′*); ^13^C-NMR (CDCl_3_, 125 MHz): *δ* 24.0 (C*H*_3_), 29.2 C-4), 34.3 (C-3), 53.7 (OC*H*_3_), 60.6 (C-2), 68.3 (C-α), 128.4 (C-4′), 128.5 (C-2′, C-6′), 128.6 (C-3′, C-5′), 136.2 (C-1′), 171.5 (C-5), 175.5 (*C*ONH_2_), 176.5 (*C*OOCH_3_); HRMS (ESI+) calcd for C_15_H_18_N_2_O_4_Na 313.1164 (M+Na)^+^ found 313.1165. **(4*S*,8a*R*)-6**: White powder; M.p. 188–189 °C; TLC (EA): R_f_ = 0.51; [α]_D_ = +87.6 (*c* 0.600; CHCl_3_/MeOH 10:3); IR (KBr): 727, 764, 1187, 1262, 1306, 1692, 1713, 2828, 3065; ^1^H-NMR (CDCl_3_, 500 MHz): *δ* 1.60 (s, 3H, C*H*_3_), 2.27 (m, 1H, H-8), 2.46–2.57 (m, 3H, H-7, H′-7, H′-8), 5.20 (s, 1H. H-4), 7.31–7.40 (m, 5H, H-Ar), 8.01 (bs, 1H, NH); ^13^C-NMR (CDCl_3_, 125 MHz): *δ* 23.5 (C*H*_3_), 28.0 (C-8), 29.2 (C-7), 60.1 (C-8a), 63.0 (C-4), 126.9 (C-2′, C-6′), 128.9 (C-4′), 129.0 (C-3′, C-5′), 135.1 (C-1′), 169.3 (C-6), 171.8 (C-1), 172.3 (C-3); HRMS (ESI-) calcd for C_14_H_13_N_2_O_3_: 257.0926 (M−H)^−^ found 257.0917.

#### 3.1.3. Synthesis of Compounds **3** and **6** by Base Induced Intramolecular Cyclocondensation

To a stirred solution of appropriate amidoester **2** or **5** in absolute EtOH (5 mL per 1 mmol of amidoester), sodium hydroxide (1 eq.) was added at room temperature. After dissolution of the hydroxide, the mixture was quenched with saturated aqueous solution of ammonium chloride (100 mL). The resulting cloudy solution was extracted with CH_2_Cl_2_ (3 × 30 mL). The combined organic phase was washed with water (20 mL), dried over anhydrous MgSO_4_, filtered and concentrated under reduced pressure. The residue was purified by FC.

*(4R,8aR)-4-Phenylperhydropyrrolo[1,2-a]pyrazine-1,3-dione* **(4*R*,8a*R*)-3a**. From **(2*R*,1*R*)-2a** (1.09 g, 4.16 mmol) and NaOH (0.17, 4.16 mmol); Recrystallization from H_2_O/MeOH; Yield 0.62 g (65%) of **(4*R*,8a*R*)-3a**. White powder; M.p 115–117 °C; [α]_D_ = +135.3 (*c* 0.823, CHCl_3_); HRMS (ESI-) calcd for C_13_H_13_N_2_O_2_: 229.0971 (M−H)^−^ found 229.0978.

*(4S,8aS)-4-Benzylperhydropyrrolo[1,2-a]pyrazine-1,3-dione* **(4*S*,8a*S*)-3b**. From **(2*S*,α*S*)-2b** (1.02 g, 3.70 mmol) and NaOH (0.15 g, 3.70 mmol); FC (PE/AcOEt 4:1 to 2:1), then recrystallization form H_2_O/MeOH; Yield 0.62 g (69%). White powder; M.p. 188–190 °C; TLC (PE/AcOEt 3:1): R_f_ = 0.20; [α]_D_ = −46.7 (*c* 0.689, CHCl_3_); IR (KBr): 700, 746, 1115, 1264, 1305, 1695, 2694, 2786, 2969; ^1^H-NMR (500 MHz, CDCl_3_): *δ* 1.78 (m, 2H, H-7, H-7′), 2.23 (m, 1H, H-8), 2.32 (m, 1H, H′-8), 2.55 (q, ^2^*J* = ^3^*J*_1_ = ^3^*J*_2_ = 8.5, 1H, H-6), 3.01 (m, 1H, H′-6), 3.07 (m, 2H, C*H*_2_), 3.84 (m, 2H, H-4, H-8a), 7.23 (d, ^3^*J* = 8.0, 2H, H-2′, H-6′), 7.26 (t, ^3^*J* = 7.5, 1H, H-4′), 7.31 (t, ^3^*J* = 7.5, 2H, H-3′, H-5′); 7.86 (bs, 1H, N*H*); ^13^C-NMR (CDCl_3_, 125 MHz): *δ* 22.5 (C-7), 27.9 (C-8), 38.1 (*C*H_2_), 52.9 (C-6), 57.2 (C-8a), 63.0 (C-4), 127.2 (C-4′), 128.8 (C-3′, C-5′), 129.3 (C-2′, C-6′), 137.4 (C-1′), 173.4 (C-3), 174.2 (C-1); HRMS (ESI+) calcd for C_14_H_16_N_2_O_2_Na: 267.1109 (M+Na)^+^ found 267.1108.

*(4R,8aR)-4-Benzylperhydropyrrolo[1,2-a]pyrazine-1,3-dione* **(4*R*,8a*R*)-3b**. From **(2*R*,1*R*)-2b** (0.51 g, 1.85 mmol) and NaOH (74 mg, 1.85 mmol); FC (PE/AcOEt 4:1 to 2:1), then recrystallization form H_2_O/MeOH; Yield 0.29 g (62%). White powder; M.p. 189–191 °C; [α]_D_ = +47.3 (*c* 0.713; CHCl_3_); HRMS (ESI+) calcd for C_14_H_16_N_2_O_2_Na: 267.1109 (M+Na)^+^ found 267.1115.

*(4S,9aS)-4-Benzylperhydropyrido[1,2-a]pyrazine-1,3-dione* **(4*S*,9a*S*)-3c**. From **(2*S*,1*S*)-2c** (1.00 g, 3.45 mmol) and NaOH (0.14 mg, 3.45 mmol); FC (PE/AcOEt 4:1 to 2:1), then recrystallization form H_2_O/MeOH; Yield 0.49 g (55%). White powder; M.p. 133 °C; TLC (PE/AcOEt 3:1): R_f_ = 0.29; [α]_D_ = −23.7 (*c* 0.667; CHCl_3_); IR (KBr): 698, 1111, 1246, 1698, 2806, 2947, 3429; 1H-NMR (CDCl3, 500 MHz): *δ* 1.45 (m, 1H, H-8), 1.52 (m, 1H, H′-8), 1.56 (m, 2H, H-7, H′-7), 1.83 (m, 1H, H-9), 2.11 (m, 1H, H′-9), 2.59 (m, 1H, H-6), 2.64 (m, 1H, H′-6), 2.98 (dd, ^2^*J* = 14.0, ^3^*J* = 6.5, 1H, H-α), 3.14 (dd, ^2^*J* = 14.0, ^3^*J* = 8.0, 1H, H′-α), 3.62 (dd, ^3^*J*_1_ = 8.0, ^3^*J*_2_ = 6.5, 1H, H-4), 3.72 (pt, ^3^J = 5.0, 1H, H-9a), 7.20-7.34 (m, 5H, H-Ar), 7.93 (bs, 1H, NH); ^1^H-NMR ((CD_3_)_2_CO), 500 MHz): *δ* 1.41 (m, 1H, H-8), 1.45-1.58 (m, 3H, H-7, H′-7, H′-8), 1.75 (m, 1H, H-9), 2.01 (m, 1H, H′-9), 2.61 (m, 1H, H-6), 2.67 (m, 1H, H′-6), 2.97 (dd, ^2^*J* = 14.0, ^3^*J* = 6.5, 1H, C*H*_2_), 3.26 (dd, ^2^*J* = 14.0, ^3^*J* = 8.0, 1H, C*H*_2_’), 3.58 (dd, ^3^*J*_1_ = 8.0, ^3^*J*_2_ = 6.5, 1H, H-4), 3.86 (pt, ^3^*J* = 5.0, 1H, H-9a), 7.18-7.34 (m, 5H, H-Ar), 9.67 (bs, 1H, N*H*); ^13^C-NMR ((CD_3_)_2_CO, 125 MHz): *δ* 22.6 (C-8), 26.2 (C-7), 26.4 (C-9), 34.4 (C-α), 52.0 (C-6) 55.4 (C-8a), 68.7 (C-4), 127.2 (C-4′), 129.1 (C-3′, C-5′), 130.2 (C-2′, C-6′), 139.5 (C-1′), 173.3 (C-3), 173.1 (C-1); HRMS (ESI+) calcd for C_15_H_18_N_2_O_2_Na: 281.1266 (M+Na)^+^ found 281.1269.

*(4R,9aR)-4-Benzylperhydropyrido[1,2-a]pyrazine-1,3-dione* **(4*R*,9a*R*)-3c**. From **(2*R*,1*R*)-2c** (0.50 g, 1.72 mmol) and NaOH (69 mg, 1.85 mmol); FC (PE/AcOEt 4:1 to 2:1), then recrystallization form H_2_O/MeOH; Yield 0.28 g (64%). White powder; M.p. 130–131 °C; [α]_D_ = +27.1 (*c* 0.493; CHCl_3_); HRMS (ESI+) calcd for C_15_H_18_N_2_O_2_Na: 281.1266 (M+Na)^+^ found 281.1269.

*(4S,8aS)-4-Methyl-4-Benzylperhydropyrrolo[1,2-a]pyrazine-1,3-dione* **(4*S*,8a*S*)-3d**. From **(2*S*,1*S*)-2d** (0.45 g, 1.55 mmol) and NaOH (62 mg, 1.55 mmol); FC (PE/AcOEt 4:1 to 2:1); Yield 0.35 g (88%). The [α]_D_ value matches this recorded for **(4*S*,8a*S*)-3d** *via* acid-mediated cyclocondensation.

*(4R,8aR)-4-Methyl-4-Benzylperhydropyrrolo[1,2-a]pyrazine-1,3-dione* **(4*R*,8a*R*)-3d**. From **(2*R*,1*R*)-2d** (0.42 g, 1.45 mmol) and NaOH (58 mg, 1.45 mmol); FC (PE/AcOEt 4:1 to 2:1); Yield 0.35 g (93%). The [α]_D_ value matches this recorded for **(4*R*,8a*R*)-3d** *via* acid-mediated cyclocondensation.

*(8aS)-4,4-Dimethylperhydropyrrolo[1,2-a]pyrazine-1,3-dione* **(8a*S*)-3f**. From **(2*S*)-2f** (0.25 g, 1.17 mmol) and NaOH (47 mg, 1.17 mmol); FC (PE/AcOEt 1:1 to 0:1); Yield 0.20 g (95%). The [α]_D_ value matches this recorded for **(8a*S*)-3f** *via* acid-mediated cyclocondensation.

*(8aR)-4,4-Dimethylperhydropyrrolo[1,2-a]pyrazine-1,3-dione* **(8a*R*)-3f**. From **(2*R*)-2f** (0.29 g, 1.36 mmol) and NaOH (54 mg, 1.36 mmol); FC (PE/AcOEt 1:1 to 0:1); Yield 0.21 g (85%). The [α]_D_ value matches this recorded for **(8a*R*)-3f** *via* acid-mediated cyclocondensation.

*(4R,8aR)-4-Phenyl-8a-methylperhydropyrrolo[1,2-a]pyrazine-1,3,6-trione (4R,8aR)-6*. From **(2*R*,α*S*)-5** (1.28 g, 4.42 mmol) and NaOH (0.18 g, 4.42 mmol); FC (PE/AcOEt 3:1 to 0:1); Yield 1.00 g (87%): 0.59 (52%) of **(4*R*,8a*R*)-6** and 0.40 g (35%) of **(4*S*,8a*R*)-6. (4*S*,8a*R*)-6**: White powder; M.p. 164–167 °C; [α]_D_ = −50.7 (*c* 0.600; CHCl_3_); HRMS (ESI-) calcd for C_14_H_13_N_2_O_3_: 257.0926 (M−H)^−^ found 257.0923.

*Methyl (2S)-1-(1-(n-butylcarbamoyl)-1,1-diphenylmethyl)-pyrrolidine-2-carboxylate* **(2*S*)-1i**. From L*-*proline (0.64 g, 5.57 mmol), benzophenone (0.85 g, 4.64 mmol) and *n*-butyl isocyanide (0.50 mL, 4.64 mmol); FC (gradient: PE/AcOEt 10:1 to 3:1): yield 0.04 g (2%). Colorless oil; TLC (PE/AcOEt 5:1): R_f_ = 0.26; [α]_D_ = −39.6 (*c* 0.833, CHCl_3_); IR (KBr): 706, 779, 1168, 1205, 1445, 1508, 1680, 1736, 2851, 2920, 2955, 3358; ^1^H-NMR (CDCl_3_, 300 MHz): *δ* 0.83 (t, ^3^*J* = 7.0, 1H, C*H*_3_), 0.86–1.04 (m, 2H), 1.09–1.40 (m, H), 1.41-1.73 (m, 4H), 2.87 (dt, ^2^*J* =^3^*J*_1_ = 11.0, ^3^*J*_1_ = 7.5, 1H, H-5), 3.13 (m, 2H), 3.34 (m, ^2^*J* = 11.0, ^3^*J*_1_ = 8.0, ^3^*J*_1_ = 5.0, 1H, H′-5), 3.72 (s, 3H, OCH_3_), 3.92 (dd, ^3^*J*_1_ = 9.0, ^3^*J*_2_ = 2.0, 1H, H-2), 7.24–7.36 (m, 6H, H-Ar), 7.37–7.44 (m, 2H, H-Ar), 7.60 (m, 2H, H-Ar), 7.76 (pt, ^3^*J* = 5.0, 1H, CON*H*); ^13^C-NMR (CDCl_3_, 75 MHz): 13.7, 19.9, 24.2, 30.7, 31.5, 39.2, 50.0 (C-5), 52.1 (O*C*H_3_), 63.2 (C-2), 79.3 (C-1), 127.17, 127.24 (C-4′, C-4″), 127.7 (C-2′, C-2″, C-6′, C-6″), 129.7, 129.9 (C-3′, C-3″, C-5′, C-5″), 139.1, 141.1 (C-1′, C-1″), 172.3 (*C*ONH), 176.9 (*C*OOCH_3_); HRMS (ESI+) calcd for C_24_H_30_N_2_O_3_Na: 417.2154 (M+Na)^+^ found 417.2148.

*Methyl (2S,1S)- and (2S,1R)-1-(1-(n-butylcarbamoyl)-1-methyl-1-phenylmethyl)-pyrrolidine-2-carboxylate* **(2*S*,1*S*)-1j** and **(2*S*,1*R*)-1j**. From L*-*proline (0.64 g, 5.57 mmol), acetophenone (0.54 mL, 4.64 mmol) and *n*-butyl isocyanide (0.50 mL, 4.64 mmol); FC (PE/AcOEt 8:1 to 3:1): yield 0.49 g (32%): 0.24 g (15%) of **(2*S*,1*R*)-1j**, 0.25 (17%) of **(2*S*,1*S*)-1j**. **(2*S*,1*S*)-1j**: Colorless oil; TLC (PE/AcOEt 5:1): R_f_ = 0.16; [α]_D_ = −29.5 (*c* 1.190, CHCl_3_); IR (KBr): 700, 1167, 1205, 1441, 1522, 1682, 1736, 2872, 2957, 3334; ^1^H-NMR (CDCl_3_, 300 MHz): *δ* 0.94 (t, ^3^*J* = 7.5, 3H, CH_2_CH_2_CH_2_C*H*_3_), 1.30–1.44 (m, 2H, C*H*_2_), 1.49-1.61 (m, 2H, C*H*_2_), 1.71 (s, 3H, C*H*_3_), 1.71-1.98 (m, 4H, H-4, H′-4, H-3, H′-3), 2.93 (pq, ^2^*J* = ^3^*J*_1_ = 9.5, ^3^*J*_2_ = 7.5, 1H, H-5), 3.11 (m, ^2^*J* = 9.5, ^3^*J*_1_ = 6.5, ^3^*J*_2_ =4.0, 1H, H′-5), 3.21–3.40 (m, 3H, H-2, C*H*_2_′′), 3.43 (s, 3H, OC*H*_3_), 7.17–7.33 (m, 3H, H-4′, H-3′, H-5′), 7.41 (d, ^3^*J* = 7.0, 2H, H-2′, H-6′), 7.91 (pt, ^3^*J*_1_ = 5.0, 1H, CON*H*); ^13^C-NMR (CDCl_3_, 75 MHz): *δ* 13.8, 17.9, 20.2, 25.2, 31.6, 31.7, 39.2, 49.5 (C-5), 51.6 (O*C*H_3_), 61.1 (C-2), 68.9 (C-1), 127.5 (C-2′, C-6′), 127.6 (C-4′), 128.1 (C-3′, C-5′), 141.2 (C-1′), 174.1 (*C*ONH), 177.0 (*C*OOCH_3_); LC/MS: 333 [M+H]^+^, retention time: 12.6 min; HRMS (ESI+) calcd for C_19_H_28_N_2_O_3_Na: 355.1998 (M+Na)^+^ found 355.1981. **(2*S*,1*R*)-1j**: Colorless oil; TLC (PE/AcOEt 5:1): R_f_ = 0.24; [α]_D_ = −34.4 (*c* 0.833, CHCl_3_); IR (KBr): 700, 764, 1167, 1209, 1443, 1522, 1682, 1736, 2870, 2945, 3369; ^1^H-NMR (CDCl_3_, 300 MHz): *δ* 0.87 (t, ^3^*J* = 7.5, 3H, CH_2_CH_2_CH_2_C*H*_3_), 1.18–1.32 (m, 2H, C*H*_2_), 1.33–1.46 (m, 2H, C*H*_2_′), 1.62–1.82 (m, 5H, H-4, H′-4, C*H*_3_), 1.89–2.05 (m, 2H, H-3, H′-3), 2.36 (m, ^2^*J* = ^3^*J*_1_ = 9.5, ^3^*J*_2_ = 7.0, 1H, H-5), 2.98 (m, ^2^*J* = 9.5, ^3^*J*_1_ = 7.5, ^3^*J*_2_ = 3.0, 1H, H′-5), 3.11 (m, 1H, C*H*′_2_′′), 3.18 (m, 1H, C*H*′_2_′′), 3.72 (s, 3H, OC*H*_3_), 3.81 (dd, ^3^*J*_1_ = 8.0, ^3^*J*_2_ = 3.5, 1H, H-2), 7.23 (t, ^3^*J* = 7.5, 1H, H-4′), 7.32 (t, ^3^*J* = 7.5, 2H, H-3′, H-5′), 7.55 (d, ^3^*J* = 7.5, 2H, H-2′, H-6′), 7.72 (pt, ^3^*J*_1_ = 5.0, 1H, CON*H*); ^13^C-NMR (CDCl_3_, 75 MHz): *δ* 13.9 (br), 17.4 (br), 20.2 (br), 24.8 (br), 31.7 (br), 32.0 (br), 39.3, 49.9, (C-5), 52.2 (O*C*H_3_), 61.4 (C-2), 69.9 (C-1), 127.3 (C-4′), 127.6 (C-2′, C-6′), 128.2 (C-3′, C-5′), 141.8 (C-1′), 174.2 (*C*ONH), 177.3 (*C*OOCH_3_); LC/MS: 333 [M+H]^+^, retention time: 15.6 min; HRMS (ESI+) calcd for C_19_H_28_N_2_O_3_Na: 355.1998 (M+Na)^+^ found 355.1990.

#### 3.1.4. Synthesis of Compounds **4** by Intramolecular U-4C-3CR Condensation

Triethylamine (1.0 eq.) was added to a cold, stirred solution of methyl (*S*)-phenylglycinate (1.0 eq.) and levulinic acid (1.0 eq.) in MeOH (100 mL), followed by *tert*-butyl isocyanide (1.0 eq.) The mixture was stirred at rt for 24 h and the volatiles were removed under reduced pressure. The residue was dissolved in DCM (100 mL) and washed with water (3 × 20 mL), brine (20 mL), dried and concentrated under reduced pressure. The residue was purified by FC.

*Methyl (2R,αS)-* and *(2S,αS)-α-(2-(tert-butylcarbamoyl)-2-methyl-5-oxopyrrolidin-1-yl)-α-phenylacetate* **(2*R*,α*S*)-4** and **(2*S*,α*S*)-4**. From methyl (2*S*)-2-phenylglycinate (3.39 g, 16.80 mmol), triethylamine (2.34 mL, 16.80 mmol), levulinic acid (1.95 g, 16.8 mmol) and *tert*-butyl isocyanide (2.0 mL, 16.80 mmol); FC (PE/AcOEt 6:1 to 1:1); Yield 5.23 g (90%): 2.70 g (47%) of **(2*S*,α*S*)-4** and 2.53 g (43%) of **(2*R*,α*S*)-4**. **(2*R*,α*S*)-4**: White powder; M.p. 160–163 °C; TLC (AcOEt): R_f_ = 0.70; [α]_D_ = –90.2 (*c* 0.7; CHCl_3_); IR (KBr): 704, 1001, 1266, 1395, 1532, 1666, 1694, 1724, 2972, 3223, 3390; ^1^H-NMR (CDCl_3_, 500 MHz): *δ* 1.26 (s, 3H, C*H*_3_), 1.41 (s, 9H, C(C*H*_3_)_3_), 1.90–2.00 (m, 1H, H-3), 2.38–2.54 (m, 2H, H′-3, H-4), 3.78 (s, 3H, OC*H*3), 4.64 (s, 1H, H-α), 7.30–7.37 (m, 6H, H-Ar), 7.91 (bs, 1H, CON*H*); ^13^C-NMR (CDCl_3_, 125 MHz): *δ* 24.0 (*C*H_3_), 28.6 (C(*C*H_3_)_3_), 29.3 (C-4), 34.4 (C-3), 51.7 (O*C*H3), 53.5 (*C*(CH_3_)_3_), 60.6 (C-2), 68.8 (C-α), 128.4 (C-4′), 128.6 (C-2′, C-6′), 128.6 (C-3′, C-5′), 136.5 (C-1′), 170.7 (C-5), 172.4 (CONH), 175.7 (*C*OOCH_3_); HRMS (ESI+) calcd for C_19_H_26_N_2_O_4_Na: 369.1790 (M+Na)^+^ found 369.1803. **(2*S*,α*S*)-4**: White powder; M.p. 162–164 °C; TLC (AcOEt): R_f_ = 0.55; [α]_D_ = −105.4 (*c* 0.7; CHCl_3_); IR (KBr): 715, 1005, 1229, 1393, 1513, 1673, 1699, 1745, 2970, 3333, 3412; TLC (EA): R_f_ = 0.55; ^1^H-NMR (CDCl_3_, 500 MHz): *δ* 1.02 (s, 9H, C(C*H*_3_)_3_), 1.41 (s, 3H, C*H*_3_), 2.11 (m, ^2^*J =* 16.0, ^3^*J*_1_ = 9.0, ^3^*J*_2_ = 7.5, 1H, H-3), 2.28 (m, ^2^*J =* 16.0, ^3^*J*_1_ = 9.5, ^3^*J*_2_ = 7.0, 1H, H′-3), 2.48 (m, 2H, H-4), 3.73 (s, 3H, OC*H*_3_), 4.84 (s, 1H, H-α), 7.26 (bs, 1H, CON*H*), 7.38 (m, 3H, H-3′, H-4′, H-5′), 7.49 (dd, ^3^*J* = 7.5, ^4^*J* = 3.5, 2H, H-2′, H-6′); ^13^C-NMR (CDCl_3_, 125 MHz): *δ* 21.9 (*C*H_3_), 27.9 (C(*C*H_3_)_3_), 28.9 (C-4), 34.8 (C-3), 51.0 (O*C*H_3_), 53.0 (*C*(CH_3_)_3_), 60.9 (C-2), 69.0 (C-α), 129.1 (C-4′), 129.2 (C-2′, C-6′), 129.8 (C-3′, C-5′), 135.5 (C-1′), 170.1 (C-5), 172.4 (*C*ONH), 177.0 (*C*OOCH_3_); HRMS (ESI+) calcd for C_19_H_26_N_2_O_4_Na: 369.1790 (M+Na)^+^ found 369.1800.

### 3.2. Pharmacological Evaluation

The compounds obtained have been submitted for *in vivo* evaluation in the Anticonvulsant Screening Program (ASP) of The National Institute of Neurological Disorders and Stroke (NINDS), Bethesda, USA [21,22]. The experiments were performed in male albino Carworth Farms No. 1 mice (weighing 18–25 g). The animals had free access to feed and water except during the actual testing period. Housing, handling and feeding were all in accordance with recommendations contained in the ‘Guide for the Care and Use of Laboratory Animals’. The test compounds were dissolved or suspended in 0.5% (v/v) aqueous solution of methylcellulose.

#### 3.2.1. The Maximal Electroshock Seizure Test (MES) [22]

In the MES test, an electrical stimulus of 0.2 s in duration (50 mA in mice) was delivered via corneal electrodes primed with an electrolyte solution containing an anesthetic agent. Mice were tested at at least two different time points (15 min, 30 min, 1 h or 4 h) following intraperitioneal administration of 100 and 300 mg/kg of test compound. Abolition of the hindlimb tonic extensor component indicated the test compound’s ability to inhibit MES-induced seizure spread.

#### 3.2.2. The subcutaneous Metrazol seizure test (scMET) [22]

The test utilized a dose of Metrazol (pentylenetetrazole, 85 mg/kg in mice). This produced clonic seizures lasting for a period of at least five seconds in 97% (CD_97_) of animals tested. At the anticipated time of testing the convulsant was administered subcutaneously. The test compound was administered intraperitoneally in mice and the animals were observed over a 30 min period. Mice were tested at at least two different time points (15 min, 30 min, 1 h or 4 h) following intraperitioneal administration of 100 and 300 mg/kg of test compound. Absence of clonic spasms indicated a compound’s ability to abolish the effect of pentylenetetrazol on seizure threshold.

#### 3.3.3. The Acute Neurological Impairment (TOX) [26]

Neurological toxicity induced by a compound was detected in mice or rats using the standardized rotorod test. Mice were tested at a minimum of two different time points (15 min, 30 min, 1 h or 4 h) following intraperitioneal administration of 100 mg/kg of test compound. Neurological impairment was demonstrated by the inability of animals to maintain equilibrium on a 6 r.p.m. rotation rod for a given time.

#### 3.3.4. The minimal clonic seizure test (6 Hz) [23,24,25]

The 6 Hz screen is an alternative electroshock paradigm that uses low-frequency (6 Hz), long-duration (3 s) electrical stimulation. Mice were tested at time intervals between 0.25 and 4 h following intraperitoneal doses of 100 mg/kg of test compound. Corneal stimulation (0.2 ms-duration monopolar rectangular pulses at 6-Hz for 3 s) was delivered by a constant-current device. During the stimulation, mice were manually restrained and released into the observation cage immediately after the current application. The seizures manifested in ‘stunned’ posture associated with rearing, forelimb, automatic movements and clonus, twitching of the vibrissae and Straub-tail. The duration of the seizure activity ranged from 60 to 120 s in untreated animals. At the end of the seizure, animals resumed their normal exploratory behavior. The experimental end point was protection against the seizure. The animal was considered to be protected if it resumed its normal exploratory behavior within 10 s from the stimulation. The quantitative determination of the median effective (ED_50_) and toxic doses (TD_50_) was conducted at previously calculated time of peak effect (TPE) using the intraperitoneal route in mice. Groups of at least eight animals were tested using different doses of test compound until at least two points were determined between 100% and 0% protection and minimal motor impairment. The dose of the candidate substance required to produce the desired endpoint in 50% of the animals in each test, and 95% confidence interval were calculated by a computer program based on methods described by Finney [27].

## 4. Conclusions

We have synthesized a series of novel stereochemically pure pyrrolo[1,2-*a*]pyrazine derivatives by use of pathways based on the U-5C-4CR and the U-4C-3CR multicomponent reactions. The compounds displayed weak to good anticonvulsant activities in the MES model, while only few of them were active in the scMET screen. The efficacy of most of the new derivatives in the 6 Hz model of pharmacoresistant partial seizures was significantly higher than in the ‘classical’ models. As a supplement to our previous extensive SAR studies within this group of compounds, we learned that: (1) increasing the distance of the benzene ring from the imide moiety of ADD408003 lowered its potency in MES, scMET and 6 Hz models, (2) introduction of non-epimerizable residue in lieu of hydrogen atom at C-4 did not enhance the pharmacological properties of the parent compound, (3) introduction of alkyl residues at C-4 produced either inactive or strongly neurotoxic and proconvulsant compounds, (4) for most of the newly synthesized derivatives, the differences in pharmacological activities between (*R*,*R*) and (*S*,*S*) were only slight.

The opposite enantiomer of ADD408003 **(4*R*,8a*R*)-3a** was most active from the series with an ED_50_ value of 47.90 mg/kg and a protective index (PI) of 5.8 in the 6 Hz model of pharmacoresistant epilepsy. The ADD408003-Levetiracetam hybrid **(4*S*,8a*S*)-6** was markedly less potent in this test, with ED_50_ value of 126.19 mg/kg and PI of 2.1. However, the overall synthetic feasibility of this compound was very high. Thus, manipulating the amino acid and keto acid component in the initial intramolecular U-4C-3CR condensation opens the access to chemical diversity of potentially interesting **(4*S*,8a*S*)-6** analogs.
